# Clinical and cost-effectiveness of the Ross procedure versus conventional aortic valve replacement in young adults

**DOI:** 10.1136/openhrt-2019-001047

**Published:** 2019-05-22

**Authors:** Howard Thom, Alexandru Ciprian Visan, Edna Keeney, Dan Mihai Dorobantu, Daniel Fudulu, Mansour T A Sharabiani, Jeff Round, Serban Constantin Stoica

**Affiliations:** 1 Bristol Medical School: Population Health Sciences, University of Bristol, Bristol, UK; 2 Cardiothoracic Surgery, Bristol Heart Institute, Bristol, UK; 3 Department of Cardiothoracic Surgery, University Hospital Southampton, Southampton, UK; 4 Cardiac Surgery, Bristol Royal Hospital for Children, Bristol, UK; 5 Cardiology, Institutul de Urgenta pentru Boli Cardiovasculare Prof Dr C C Iliescu, Bucuresti, Romania; 6 National Heart and Lung Institute, Imperial College London, London, UK; 7 Institute of Health Economics, Edmonton, Alberta, Canada

**Keywords:** ross procedure, autograft aortic valve replacement, mechanical avr, bioprosthesis avr, clinical trial, outcomes, economical analysis

## Abstract

**Objectives:**

In young and middle-aged adults, there are three current options for aortic valve replacement (AVR), namely mechanical AVR (mechAVR), tissue AVR (biological AVR) and the Ross operation, with no clear guidance on the best option. We aim to compare the clinical effectiveness and cost-effectiveness of the Ross procedure with conventional AVR in young and middle-aged adults.

**Methods:**

This is a systematic literature review and meta-analysis of AVR options. Markov multistate model was adopted to compare cost-effectiveness. Lifetime costs, quality-adjusted life years (QALYs), net monetary benefit (NMB), population expected value of perfect information (EVPI) and expected value of partial perfect information were estimated.

**Results:**

We identified 48 cohorts with a total number of 12 975 patients (mean age 44.5 years, mean follow-up 7.1 years). Mortality, bleeding and thromboembolic events over the follow-up period were lowest after the Ross operation, compared with mechAVR and biological AVR (p<0.001). Aortic reoperation rates were lower after Ross compared with biological AVR, but slightly higher when compared with mechAVR (p<0.001). At a willingness-to-pay threshold of £20effective. At a willingness-to-pay threshold of £20, 000 per QALY000 per QALY, the Ross procedure is more cost-effective compared the Ross procedure is more cost-effective compared withwith conventional AVR, with a lifetime incremental NMB of £60 conventional AVR, with a lifetime incremental NMB of £60 952 (952 (££3030 236236 to to ££7979 464). Incremental costs were £12464). Incremental costs were £12 323 (323 (££61086108 to to ££1515 972) and incremental QALYs 3.66 (1.81972) and incremental QALYs 3.66 (1.81 to to 4.76). The population EVPI indicates that a trial costing up to £2.03 million could be cost 4.76). The population EVPI indicates that a trial costing up to £2.03 million could be cost--effective.

At a willingness-to-pay threshold of £20 000 per QALY, the Ross procedure is more cost-effective compared with conventional AVR, with a lifetime incremental NMB of £60 952 (£30 236 to £79 464). Incremental costs were £12 323 (£6108 to £15 972) and incremental QALYs 3.66 (1.81 to 4.76). The population EVPI indicates that a trial costing up to £2.03 million could be cost-effective.

**Conclusions:**

In young and middle-aged adults with aortic valve disease, the Ross procedure may confer greater quality of life and be more cost-effective than conventional AVR. A high-quality randomised trial could be warranted and cost-effective.

**Key questions**

**What is already known about this subject?**

- The Ross operation has recently shown favourable outcomes compared with other aortic valve replacement options in young adults.

**What does this study add?**

- In this study we have found that the Ross operation has better outcomes, such as mortality, freedom from reintervention or freedom from embolic events, and is more economically efficient in the long term compared with conventional aortic valve replacement in the young.
- Additionally, we propose that a comparative clinical trial with a budget of £2 million would be warranted and cost-effective.

**How might this impact on clinical practice?**

- Young patients who undergo conventional aortic valve replacement, such as with mechanical or biological prosthesis, face either lifelong anticoagulation and embolic risks, or rapid valve degeneration and reinterventions, respectively.
- The Ross procedure is re-emerging as a better option in this age group.
- Lower overall costs reflect these better outcomes, but available data are only retrospective.
- A randomised clinical trial, shown in this study to be economically efficient, is planned to directly compare aortic valve replacement options in non-elderly patients.

## Introduction

The standard treatment for aortic valve (AoV) disease is aortic valve replacement (AVR) when repair is not possible.^1^ Options include mechanical (mechAVR) and biological (biological AVR) prostheses, or conventional AVR (cAVR) and the Ross procedure (pulmonary autograft), and rarely homograft valves. Bioprostheses are durable in older patients,^2^ with the survival advantage of mechAVR over biological AVR disappearing around the age of 60.^3^ However, decision making in young and middle-aged adults is challenging, with a more marked impact of cAVR on survival in younger patients,^4^ and with biological AVR and mechAVR being associated with reduced life expectancy.^5^ Furthermore, there are issues with biological valve degeneration and mechanical valve need for anticoagulation, which are more important in young patients.

Critics of the Ross procedure point out that it creates a ‘two-valve disease’. However, multiple studies show very good early results and better long-term survival, comparable with that of the general population.^6–8^ The Ross procedure affords superior haemodynamics in the autograft^9^ and typically uses a homograft in the lower pressure pulmonary circulation. Despite better outcomes, the Ross procedure is underused, partly due to technical complexity. However, it can be performed safely in congenital centres and hospitals where complex aortic root operations are routine.^10^

Cost-effectiveness analysis compares the costs and effects of treatments, usually measured as quality-adjusted life years (QALYs), on a monetary scale and thus aids reimbursement decision making by healthcare payers.^11^ To date there have been no cost-effectiveness analyses evaluating the Ross procedure as an option for aortic stenosis (AS) in young and middle-aged adults. Previous economic evaluations have compared cAVR and transcatheter aortic valve implantation (TAVI) with each other and with medical management.^12–14^ The results have been variable with respect to the cost-effectiveness of TAVI compared with cAVR in high-risk patients, both being cost-effective compared with medical management.^13–15^ Value of information analysis is a method to use the outputs of cost-effectiveness analyses to prioritise and design research.^11^ Only one publication, comparing TAVI and medical management, has applied value of information analysis to AS.^16^

Using meta-analysis, we compared the clinical effectiveness of the Ross procedure with biological AVR and mechAVR for the treatment of young and middle-aged adults with AS. We then used these results to inform a comprehensive cost-effectiveness comparison of these procedures in England and Wales from a National Health Service (NHS) perspective. Another objective is to estimate the value of information of a randomised controlled trial comparing cAVR with the Ross procedure.

## Methods

Our model structure and inputs were informed by a review of published cost-effectiveness models evaluating cAVR and other AoV procedures. Detailed methods and results are presented in online supplementary material A. In brief, we identified 12 papers published between 2012 and 2016 comparing cAVR, TAVI and medical management. As these models did not include the Ross procedure, we adapted their structures to capture important features specific to Ross. Input parameters, not identified in the clinical or economic systematic reviews, were informed by targeted literature searches.

10.1136/openhrt-2019-001047.supp1 Supplementary data

### Model structure and inputs

Detailed description of the model structure and the evidence base for its inputs is provided in online supplementary material B. Briefly, we adopted a discrete-time Markov multistate model, a common choice in cost-effectiveness analysis^17^ and in aortic stenosis.^12–14^ The model structure is illustrated and explained in online supplementary material figures S1 and S2. Patients enter the model following an initial Ross or cAVR procedure, and their states and events determine costs and QALYs per cycle. We adopt an NHS costing perspective and lifetime time horizon with a 1-year cycle length, which is consistent with previously published models.^18–20^

**Figure 1:**
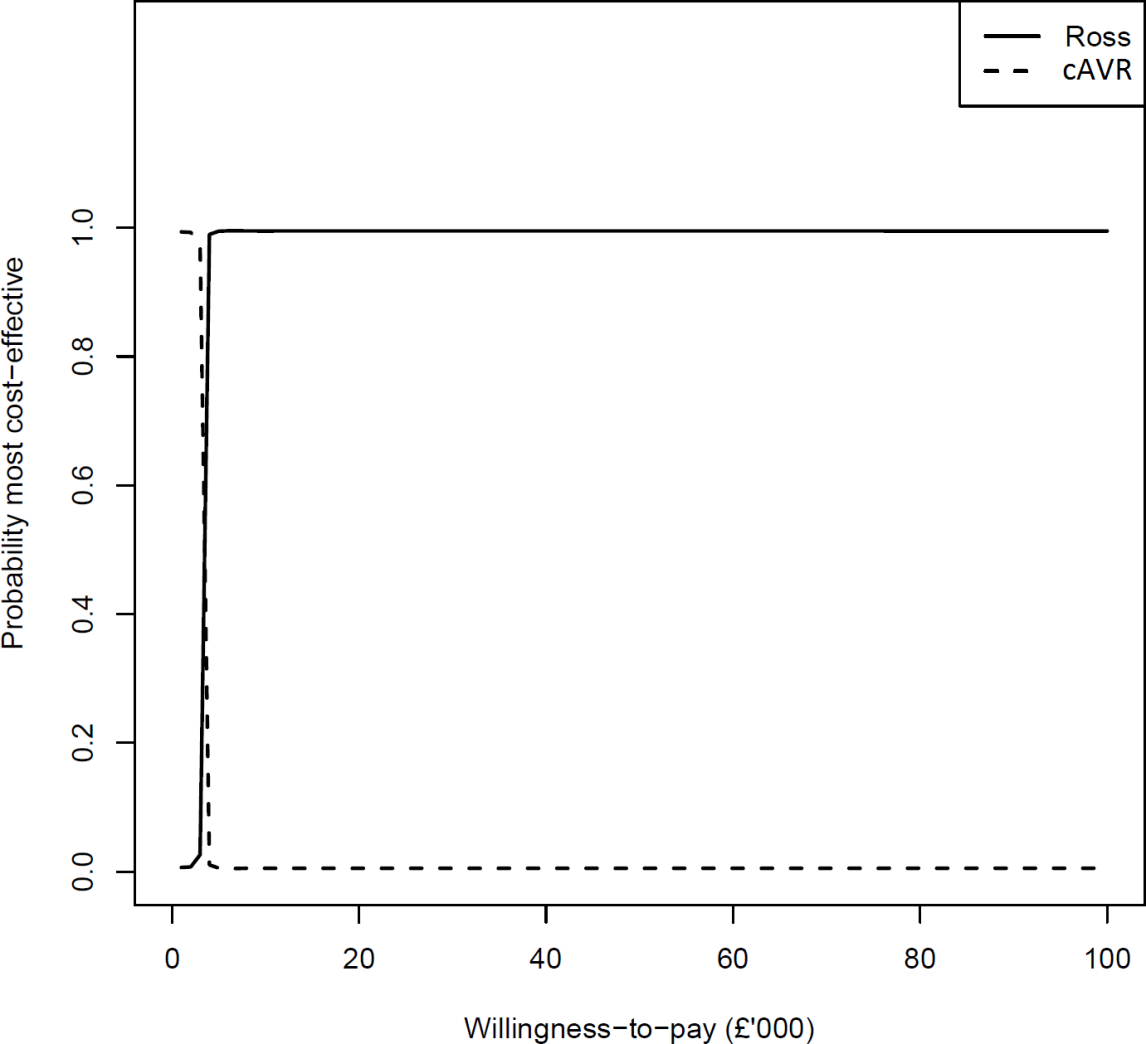
Cost-effectiveness acceptability curve.

We estimated the probabilities of events and transitions using a systematic literature review and meta-analysis, detailed in online supplementary material C. We searched MEDLINE, EMBASE and Cochrane Library for studies published between January 1990 and January 2016 in adults aged 18–65. A Preferred Reporting Items for Systematic Reviews and Meta-Analyses flow chart is included in online supplementary figures S3. Data from 41 observational studies and 2 clinical trials were pooled using a random-effects meta-analysis model (online supplementary figures S4-S12 and table S2).

Several sources were used to estimate the state occupancy and event costs and utilities (online supplementary table S1), including evidence from the extensive literature on stroke prevention in atrial fibrillation.^21–24^ Costs of the cAVR and Ross procedure, and of other one-off events, were estimated according to the 2016/2017 NHS tariff. Cost estimates were assumed fixed as they represent agreed prices paid to hospitals for activity. The healthy state utility followed that of Doble *et al*,^13^ while utilities for other states and disutilities for events were adapted from the atrial fibrillation literature.^24 25^ We averaged mechAVR and biological AVR probabilities using an estimated 0.23 proportion of cAVR that is biological, with further details in online supplementary material B (table S2).

### Cost-effectiveness outputs

We estimated lifetime costs and QALYs for patients undergoing cAVR and Ross procedure, as well as the net monetary benefit (NMB) and incremental NMB (INMB). The NMB is a summary of both costs and QALYs on the monetary scale; it is calculated by subtracting costs from monetary benefit, given by multiplying QALYs by a willingness-to-pay threshold, commonly assumed to be £20 000 for the UK NHS,^26^ but often up to $100 000 in the USA.^27^ We performed probabilistic sensitivity analysis (PSA) to simulate 10 000 lifetime costs, QALYs, NMBs and INMBs. Means and 95% Bayesian credible intervals (CrI) were estimated for all quantities.^11^ We generated a cost-effectiveness acceptability curve (CEAC), which plots the probability of Ross procedure or cAVR being most cost-effective for a range of willingness-to-pay thresholds.^26^

### Value of information analysis

We estimated the per-person expected value of perfect information (EVPI), which is the expected improvement in decision making, or value, from removing all uncertainty in all parameters.^11^ We used multilevel Monte Carlo to estimate per-person expected value of partial perfect information (EVPPI), the expected value of removing all uncertainty in only a subset of parameters.^28^ We focused on sets of parameters likely to be informed by studies comparing cAVR with the Ross procedure. We scaled to per-population EVPI and EVPPI using a 3.5% per annum discounted 25-year technology horizon population size of 16 376 (details in online supplementary material B).

### Deterministic sensitivity analyses

We conducted a one-way sensitivity analysis comparing Ross procedure with cohorts treated solely with either biological AVR or mechAVR, rather than the mixture of our base case. Also, as some costs and utility parameters were fixed, these would not be included in our primary sensitivity analyses of PSA and EVPI. These 15 parameters are listed in online supplementary material B. We conducted one-way sensitivity analyses where these parameters were set to 50% and 150% of their values.

## Results

### Epidemiological meta-analysis results

Estimated probabilities are summarised in table 1, with forest plots detailed in online supplementary material figures S4-S12. Mortality over the follow-up period was lower in Ross patients when compared with mechAVR and biological AVR (0.54%/year vs 1.38%/year and 2.54%/year, respectively; p<0.001). There was no evidence of difference in early mortality between groups. The Ross procedure was associated with lower bleeding rates compared with mechAVR and biological AVR (0.11%/year vs 0.69%/year and 0.31%/year, respectively; p<0.001). The number of thromboembolic events was also lower following the Ross procedure in comparison with mechAVR and biological AVR (0.26%/year vs 0.86%/year and 0.57%/year, respectively; p<0.001). Reoperation rates on the AoV were reduced in Ross patients when compared with biological AVR, but slightly higher when compared with mechAVR (0.54%/year vs 1.28%/year and 0.37%/year, respectively; p<0.001). No significant differences were found in terms of infective endocarditis.

**Table 1:** Comparison of outcomes between the Ross operation, mechAVR and biological AVR: results from the meta-analysis

	Cohorts	Ross	mechAVR	biological AVR	P value
Early mortality (%)	46	0.24 (0.17 to 0.34)	0.3 (0.19 to 0.45)	0.28 (0.16 to 0.48)	0.75
Total mortality (%/year)	47	0.54 (0.45 to 0.64)	1.38 (1.11 to 1.74)	2.54 (1.8 to 3.59)	<0.001
AoV reoperation (%/year)	47	0.54 (0.42 to 0.69)	0.37 (0.27 to 0.5)	1.28 (0.88 to 1.85)	<0.001
Bleeding (%/year)	44	0.11 (0.06 to 0.20)	0.69 (0.5 to 0.96)	0.31 (0.14 to 0.69)	<0.001
Thromboembolic events (%/year)	44	0.26 (0.19 to 0.37)	0.86 (0.62 to 1.18)	0.57 (0.39 to 0.84)	<0.001
AoV reoperations for IE (%/year)	44	0.16 (0.12 to 0.2)	0.17 (0.1 to 0.27)	0.26 (0.18 to 0.39)	0.08
Conservatively treated IE (%/year)	46	0.21 (0.15 to 0.3)	0.27 (0.18 to 0.42)	0.29 (0.1 to 0.87)	0.64
Total IE (%/year)	39	0.34 (0.27 to 0.42)	0.44 (0.3 to 0.64)	0.63 (0.32 to 1.22)	0.14
RVOT reinterventions (%/year)	22	0.43 (0.34 to 0.54)			

Data are from random-effects models. Values in brackets are 95% CI.

AoV, aortic valve;IE, infective endocarditis;RVOT, right ventricular outflow tract; biological AVR, tissue aortic valve replacement;mechAVR, mechanical aortic valve replacement.

### Cost-effectiveness results

Estimated means and 95% CrI from the cost-effectiveness analysis are summarised in table 2. The expected lifetime incremental net benefit of £60 952 (£30 236 to £79 464) and its narrow CrI which excludes zero suggest there is strong evidence that the Ross procedure is more cost-effective than cAVR at a willingness-to-pay threshold of £20 000. These findings were repeated at a willingness-to-pay threshold of £50 000. There is also strong evidence that the Ross procedure is associated with higher lifetime QALYs, having an incremental QALY of 3.66 (1.81 to 4.76), and thus superior effectiveness to cAVR. Although the Ross procedure has higher lifetime costs than cAVR, with incremental costs of £12 323 (£6108 to £15 972), this is outweighed by the larger benefit in QALYs.

**Table 2:** Comparison of cost-effectiveness between the Ross operation and cAVR

	cAVR	Ross	Incremental values Ross vs AVR
Costs	£33 812 (£31 028 to £39 845)	£46 135 (£44 267 to £48 263)	£12 323 (£6108 to £15 972)
QALYs	11.5 (10.4 to 13.3)	15.2 (14.4 to 15.9)	3.66 (1.81 to 4.76)
NMB at £20 000	£196 385 (£177 231 to £227 366)	£257 337 (£241 933 to £270 844)	£60 952 (£30 236 to £79 464)
NMB at £50 000	£541 679 (£490 382 to £627 964)	£712 545 (£672 962 to £748 287)	£170 866 (£84 507 to £222 263)

Values represent means and CI in brackets.

cAVR, conventional aortic valve replacement;NMB, net monetary benefit; QALYs, quality-adjusted life year.

The limited uncertainty in our decision is illustrated by the CEAC (figure 1), where it appears that beyond a very low willingness-to-pay threshold (approximately £3000), there is almost 100% probability that the Ross procedure is the most cost-effective treatment option. The Ross procedure would be most cost-effective for all willingness-to-pay thresholds above £3000 per QALY, including £20 000, £50 000 and £100 000.

The deterministic sensitivity analyses showed no impact on the results or conclusions of varying the 15 parameters. The Ross procedure remained cost-effective at a willingness-to-pay threshold below £6000 for all sensitivities. Sensitivity analysis found the Ross procedure to be superior to biological AVR and mechAVR alone; this sensitivity also indicated that mechAVR was superior on costs and QALYs to biological AVR, in line with existing literature.^29^

### Value of information results

The estimated per-person expected value of information (EVPI) at willingness-to-pay £20 000 was £123.70 and 25-year population EVPI of £2.03 million. This suggests that the proposed trial design costing up to £2.03 million could be cost-effective. The EVPPI results suggest that although studies on the utilities, poststroke disability costs and events informed by the meta-analysis could offer value of up to £425 000, £342 000 and £307 000, a value of up to £1.90 million could come from studying only the early event rates of stroke and bleed immediately following cAVR or Ross procedure. A trial comparing Ross and cAVR and informing the meta-analysis, early events and other epidemiological parameters, but not costs or utilities, could approach the maximum value of £2.03 million for decision making. Value of information results are detailed in table 3.

**Table 3:** EVPI and EVPPI for input parameters to the cost-effectiveness model

Parameters	Individual EVPPI	Population EVPPI*
Total EVPI	123.7 (118, 129.4)	2 026 008 (1 932 365; 2 119 651)
Meta-analysis	18.75 (3.887, 33.62)	307 068 (63 655; 550 481)
Utilities	25.97 (11.11, 40.83)	425 290 (181 877; 668 702)
Poststroke disability costs	20.94 (6.078, 35.81)	342 953 (99 541; 586 366)
Bleed or stroke following cAVR or Ross†	116.1 (101.2, 130.9)	1 900 959 (1 657 546; 2 144 372)
Other epidemiological parameters‡	31.15 (16.28, 46.01)	510 077 (266 665; 753 490)
Ross trial (no costs or utilities)§	123.7 (108.9, 138.6)	2 026 008 (1 782 596; 2 269 421)

*Population EVPPI given by multiplying individual EVPPI by 8263 population size.

†Early events of stroke or bleed following any of biological AVR, mechAVR or Ross procedure.

‡Other epidemiological parameters are probabilities of disability following stroke; stroke following reoperations for IE on AoV, reoperations for any cause on AoV, reoperations on the pulmonary valve; death following stroke, bleeding events, conservatively treated IE, reoperations for IE on AoV, reoperations for any cause on AoV, reinterventions on the pulmonary valve.

§Ross trial informing all parameters of the meta-analysis, bleed or stroke immediately following cAVR or Ross procedure, and other epidemiological parameters but not costs and utilities.

AoV, aortic valve; EVPI, expected value of perfect information; EVPPI, expected value of partial perfect information; IE, infective endocarditis; biological AVR, tissue aortic valve replacement; cAVR, conventional aortic valve replacement; mechAVR, mechanical aortic valve replacement.

## Discussion

The results of our meta-analysis show lower mortality and fewer thromboembolic events for the Ross procedure versus cAVR. Rates of surgical reoperations on the AoV were lower than for biological AVR but higher than in mechAVR. Our cost-effectiveness analysis found that the Ross procedure would be the most cost-effective option for all willingness-to-pay thresholds above £3000 per QALY, due to the greater incremental effectiveness, despite the higher lifetime estimated costs.

We also conducted value of information analyses: a trial looking only at epidemiological parameters and costing up to £2.03 million could offer value for money. Our results indicated that most of the important uncertainty lies in the expected numbers of bleeds and strokes immediately following cAVR or Ross surgery; a trial estimating only these rates could be worth up to £1.90 million to decision makers. Limited value of £300 000–£425 000 was found for smaller study designs that measured utilities, poststroke disability cost or general event rates.

Our meta-analysis is in line with other recent findings, namely by Mazine *et al*^30^ and McClure *et al*.^31^ Both found that the Ross procedure had generally better outcomes compared with biological AVR or mechAVR. McClure *et al*^31^ found lower thromboembolic events and better health-related quality of life for the Ross procedure, but neither meta-analysis considered cost-effectiveness generally or the value of further research.

As we rely on UK NHS costs, our cost-effectiveness and value of information results are specific to that perspective. However, the results of our meta-analysis and our modelling results of 15.2 (14.4 to 15.9) QALYs on the Ross procedure compared with 11.5 (10.4 to 13.3) QALYs on cAVR are potentially generalisable. To adapt to other countries, costs of events and management would need to be changed. However, when developing our model structure, we referred to models developed in the USA, Canada and Belgium,^13 18 32^ meaning it is not specific to the UK NHS. We would be willing to share our structure and software code with interested researchers to develop country-specific adaptations.

These results come at a time when the Ross operation is under much scrutiny, with several new studies reporting excellent long-term outcomes with this technique, achieving survival comparable with that of the general population.^6 7^ Several authors have suggested that the current guidelines regarding AVR in the young should be reconsidered with these new data in mind.^33 34^

On the other hand, tissue valves are associated with poor results in the young, as shown in our meta-analysis, while mechanical valves bring the challenge of anticoagulation. Young patients, especially women planning a pregnancy, are reluctant to accept it, as it negatively impacts quality of life.^35^ In addition, there is a cumulative risk of bleeding and thromboembolic events, shown to be significantly lower for Ross patients when compared with mechAVR.^10^ In our meta-analysis bleeding or thromboembolic events were four times more frequent after mechAVR, no doubt due to anticoagulation, with a non-negligible cumulative 1.55% rate per year. In younger, more active patients, the promise of fewer restrictions and lower risk of disability might overshadow the need for more reinterventions.

Given the more complex nature of the procedure, with more reinterventions being performed, there is the issue of the total burden on the health system, and whether the Ross procedure offers a sufficient advantage in quality of life to compensate for the added costs. Our economic analysis has shown that, although it has higher initial costs, the Ross procedure achieves a significant net benefit in the long term, no doubt through the significantly lower incidence of stroke.

At present, for the young patients with AoV disease, the European guidelines do not mention the Ross procedure at all, while the American ones offer it as an alternative when anticoagulation is difficult to achieve.^36^ The accumulated evidence in the past few years strongly suggests that the benefits from the Ross procedures outweigh the disadvantages in this group. Our study enforces this notion and adds the cost-effectiveness dimension. A clinical trial already exists comparing the Ross procedure with aortic homograft,^8^ but currently the main question is how it fares when tested against mechAVR and new-generation bioprostheses. A clinical trial to answer that question is warranted, with sufficient data coming from registries to support it; we have shown that even at a budget of over £1 million, it would still prove value for money to the health service providers.

### Limitations

To design a computationally practical model, we did not include every adverse event associated with AVR. We excluded renal failure, pacemaker, arrhythmia and myocardial infarction, which were included in previous models,^12–15^ although these are less relevant in non-elderly patients. Our model recorded only history of the most serious event. These limitations could be overcome by expanding the number of model states, as in atrial fibrillation models,^24^ or by including tunnel states,^26^ but this could render EVPPI analysis computationally infeasible.^37^ Adopting an NHS rather than a societal perspective limits our model’s utility. As the Ross procedure is associated with a lower stroke and reoperation rate than biological AVR, including societal costs would likely increase its cost-effectiveness. Although there was limited uncertainty in our results, there were few high-quality studies available on many epidemiological parameters and utilities, which we represented with wide uncertainty margins. Further limitations arise from the meta-analysis used to inform the parameters and utilities of the economic model. This was primarily based on observational studies, most of them retrospective. Selection criteria were used, and unpublished data, abstracts and presentations were excluded, generating a selection bias. There is also an inherent bias in the centres used for such studies, as the requirement for expertise in the Ross procedure limits the choice of centres. Finally, we have not estimated the expected value of sample information (EVSI). EVPI and EVPPI provide only an upper bound on trial value, while EVSI values specific trial designs.^37 38^ There is also substantial uncertainty about the number of Ross-eligible patients per year; our estimate of 960 patients per year in the UK was based on extrapolations from the existing evidence, although we were conservative in our estimates.

## Conclusions

In young patients needing AVR, the Ross operation achieves better long-term survival, fewer bleeding and thromboembolic events, at the cost of more reinterventions, including those on the right ventricular outflow tract. Furthermore, our economic analysis showed that the Ross procedure is more cost-effective compared with cAVR. Despite this, the Ross operation does not have a clear place in guidelines, in lack of a good-quality clinical trial. Our study found that a trial comparing these options costing up to £2 million could offer value for money. An international collaboration started to generate this evidence prospectively and the trial is now in the pilot phase.
