# Correction: *Clinical and cost-effectiveness of the Ross procedure versus conventional aortic valve replacement in young adults*

**DOI:** 10.1136/openhrt-2019-001047corr1

**Published:** 2019-06-18

Thom H, Visan AC, Keeney E, *et al.* Clinical and cost-effectiveness of the Ross procedure versus conventional aortic valve replacement in young adults. *Open Heart* 2019;6:e001047. doi:10.0036/openhrt-2019-001047

The authors want to alert readers to the following error identified in the published version.

The co-author name Alexandru Ciprian Ciprian Visan has been corrected to Alexandru Ciprian Visan

