# Supplementary material for: Clinical and cost-effectiveness of the Ross procedure versus conventional aortic valve replacement in young adults
Source: Open Heart. 2019 May 22;6(1):e001047. doi: 10.1136/openhrt-2019-001047 (PMC6546187; doi:10.1136/openhrt-2019-001047)
Supplement: Supplementary data [file openhrt-2019-001047supp001.docx]

**SUPPLEMENTAL MATERIAL A**

**SYSTEMATIC REVIEW ON COST-EFFECTIVENESS OF SURGICAL AORTIC VALVE REPLACEMENT OR IMPLANTATION TECHNIQUES.**

The systematic review of economic literature is broader in scope than the clinical review. The aim of the review is to identify existing research on the cost-effectiveness of surgical aortic valve replacement or implantation techniques. This may or may not include pulmonary autograft (the Ross procedure). Common methods for replacement or implantation include transcatheter aortic valve implantation (TAVI) and surgical aortic valve replacement (sAVR).

The economics element of the review is not limited by age. One of the review aims was to identify any previous economic models for purposes of evaluating modelling methodology. These could be in any population group. A number of studies were identified in the scoping review that were comparisons of minimally invasive access methods compared with sternotomy. While these sometimes are wholly or partially in patients having aortic valve replacement, they are not comparisons of aortic valve replacement techniques and so are exlcuded.

**Search strategy**

**Embase and Medline**

Embase and Medline were searched using the OVID interface. The search was restricted to post 1990 studies, in English. The economics search 1^st^ used the same terms as usedin the clinical search, returning 8600 results (including duplicate results from across the Medline and Emabse databases). An economics filter was then applied to the clinical search results. 110 publications were identified after applying the economic filter. This was reduced to 79 studies after removal of duplicates. These were then screened by title and abstract.

Inclusion criteria

- Economic evaluations of aortic valve replacement using pulmonary autograft (the Ross method) compared to at least one other treatment

Exlcusion criteria

- Studies that were not proper economic evaluations (2 or more comparators, cost per effect etc) were excluded

7 papers were retreived from these results for full consideration for inclusion in the review.

**NHS EED.**

In addition to Medline and Embase, a search was conducted of the NHS EED specialist database of economic evaluations, maintained by the Centre for Reviews and Dissemination, University of York. This database contains records of economic evaluations published up until 2015. The search functionality is highly limited and so a very sensitive approach was used. Search terms were as broad as possible to detect the greatest number of potentially relevant studies. The following terms were used:

- Valve AND replacement (52 records identified)
- Valve AND implantation (39 records identified)

It is not possible to automatically remove duplicates from individual NHS EED search results using the NHS EED web interface, and so both sets of results were screened individually. After screening each set of abstracts and removal of any duplicates in the remaining abstracts, a total of 16 records were identified. NHS EED results were then compared with the results of the title and abstract screening from the Pubmed and Embase searches to identify any additional duplicates. No duplicates were identified and removed from the search results.

**Overall search results**

A total of 23 records were identified for retrieval and full assessment for inclusion in the review. 12 papers were deemed to meet the inclusion criteria. The following data was extracted from each article:

- Author
- Year
- Title
- Intervention(s)
- Comparator
- Target population
- Perspective
- Model type
- Time Horizon
- States in model
- Events
- Rate of events
- Efficacy
- Data source
- Costs included
- Cost sources
- Utility sources
- Results
- Conclusion
- Whether VOI analysis was completed

The aim of the review was to identify existing models to inform the development of the model of the Ross procedure, and no formal synthesis of the evidence was planned.

**Search terms**

**Clinical:**  (exp HEART VALVE REPLACEMENT/ OR heart valve implantation.mp. OR aortic valve replacement.mp. OR heart valve insertion.mp. OR exp AORTA VALVE REPLACEMENT/) AND (exp ROSS PROCEDURE/ OR pulmonary autograft.mp. OR exp HEART VALVE PROSTHESIS/ OR mechanical valve.mp. OR AUTOGRAFT/ OR tissue valve.mp. OR biological valve.mp. OR BIOPROSTHESIS/ OR XENOGRAFT/ OR porcine.mp.) limit to (english language and embase and yr="1990 -Current" and (adolescent <13 to 17 years> or adult <18 to 64 years>))

The above terms were combined with filter terms for identifying economic studies from the Centre for Reviews and Dissemination.

**Economic**: economics/ exp "costs and cost analysis"/ economics, dental/ exp "economics, hospital"/ economics, medical/ economics, nursing/ economics, pharmaceutical/ (economic$ or cost or costs or costly or costing or price or prices or pricing or pharmacoeconomic$).ti,ab. (expenditure$ not energy).ti,ab. value for money.ti,ab. budget$.ti,ab. or/1-11 ((energy or oxygen) adj cost).ti,ab. (metabolic adj cost).ti,ab. ((energy or oxygen) adj expenditure).ti,ab. or/13-15 12 not 16 letter.pt. editorial.pt. historical article.pt. or/18-20 17 not 21 Animals/ Humans/ 23 not (23 and 24) 22 not 25

***SUPPLEMENTAL MATERIAL B***

***ECONOMIC MODEL***

***Additional details of the cost-effectiveness model***

***Model structure***

We adopted a discrete-time Markov multistate model, which is among the most common model class in cost-effectiveness analysis ^1, 2^ and in AS ^3-8^. The model structure is illustrated and explained in Figure S1. This includes a ‘Healthy’ no complication state, a ‘Death’ state, plus three complication states: (extra-cranial, internal) bleed, (haemorrhagic or ischemic) stroke with no disability, or stroke with disability. These conditions have long-term impacts on quality of life and costs. Patients enter the model following an initial Ross or AVR procedure with some probability of beginning in one of the five states. Patient cohorts transition at the end of each model cycle; patients occupy only one state per cycle and this determines the costs and QALYs accumulated for that cycle. We adopt an NHS costing perspective and lifetime time horizon with a 1-year cycle length, which is consistent with previously published models ^8-10^.

In addition to the four primary events with long-term consequences our model includes six transient events with only a one-off disutility and/or cost: infective endocarditis (IE) treated conservatively, reoperation on the aortic valve (AoV) for IE, reoperation on the AoV for other complications, reoperation on the PV, catheter-based procedures on the PV, and concomitant reoperations on the AoV and PV. These transient events act as pathways between health states (Figure S2) and it is assumed they have no long-term consequences on quality of life, costs, or event risks.

| **Supplementary Figure S1.** Markov model following Ross or conventional AVR* |
| --- |
| 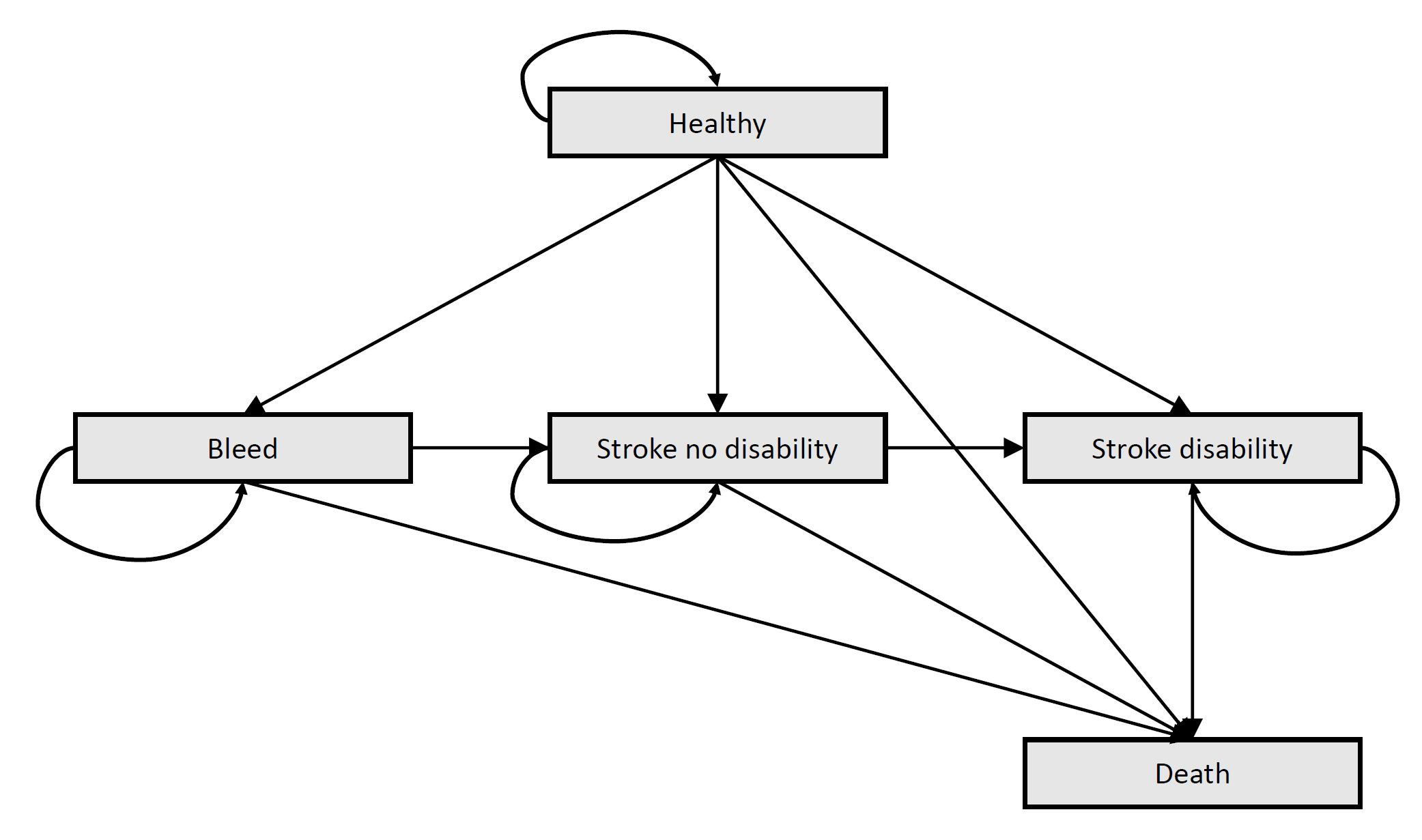  *Health states represent the history of events, rather than the current event. Patients who have experienced at least one bleed in the past occupy the bleed state, and similarly for disabling and non-disabling stroke. Patients can also experience one of 6 transient events. Patients in all states, except death, can experience at most one of any of the 10 events each cycle but will only change state if the event is up the hierarchy of bleed, stroke no disability, stroke disability, and death. |

| **Supplementary Figure S2.** Transition pathways between states via transient events in the Markov model* |
| --- |
| 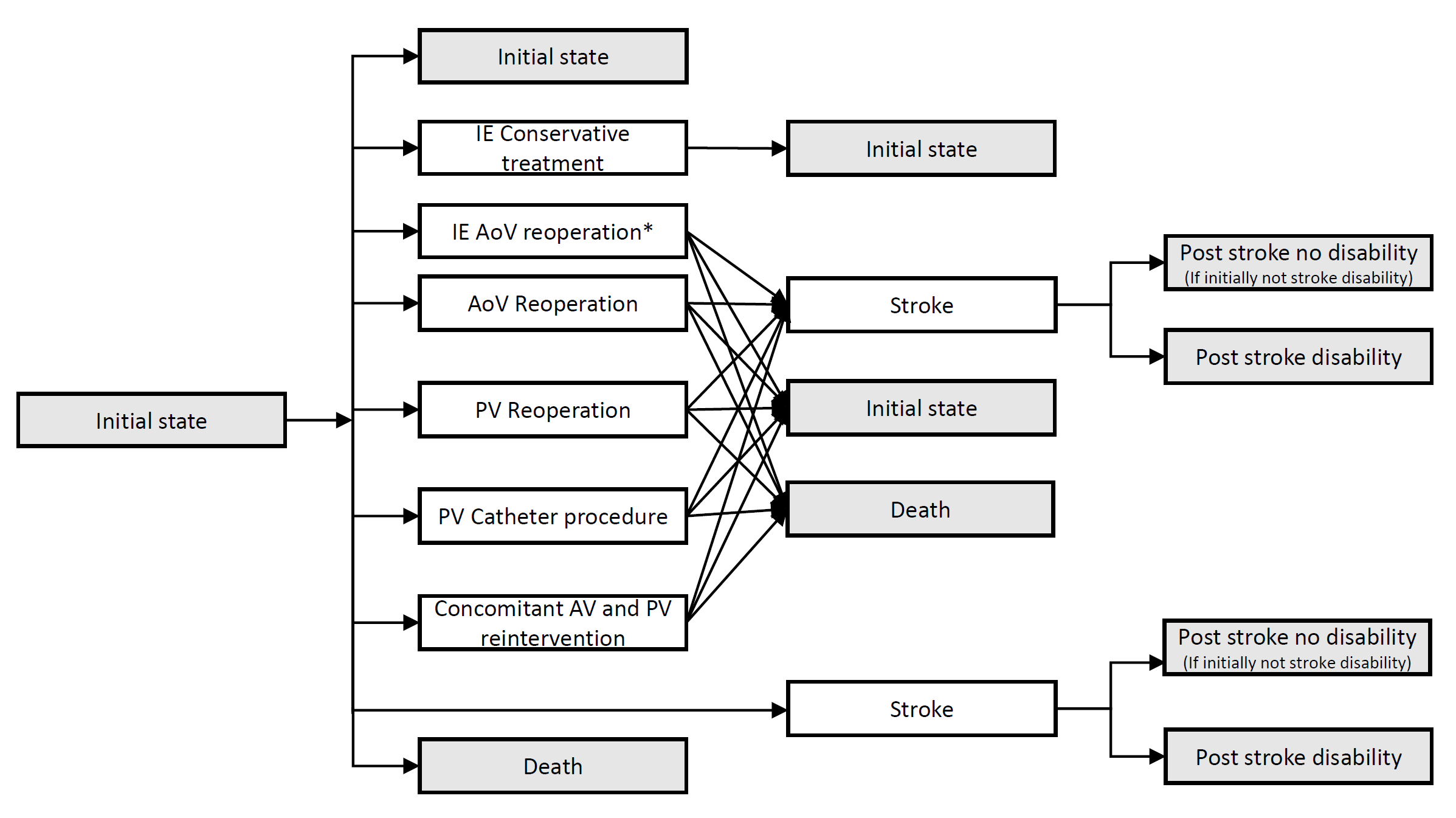  *Initial state can be healthy, bleed, post stroke no disability, post stroke disability. A patient returns to the initial state unless they experience a stroke or die along the pathway. |

***Epidemiological model inputs***

Our cost-effectiveness model requires probabilities of the four events with long-term healthcare consequences and six transient events in patients who have undergone Ross or AVR procedures. To obtain these estimates we conducted a systematic literature review and meta-analyses. We searched the MEDLINE, EMBASE and the Cochrane Library for studies published between January 1990 and January 2016 in adults aged 18-65. This generated 8,370 studies for screening and assessment by full text. Publications were rated for inclusion by a second independent reviewer. Data from 41 observational studies and 2 clinical trials were then pooled using a random effects meta-analysis model. These studies covered 48 cohorts with a total number of 12,975 patients, with a mean age of 44.5 years and a mean follow-up of 7.1 years (details in Table S1).

As initialising our Markov model requires the probabilities of experiencing bleed or stroke immediately following Ross or conventional AVR, we conducted a random effects meta-analysis of studies reporting short-term (<30 day) complications (Table S2). We further estimated probabilities of consequences of events using studies identified in the systematic literature review (details in Supplemental C).

**Proportion AVR that is biological and mechanical.**

To combine the tissue and mechanical AVR events probabilities, we needed the proportion of AVR that is of each type. Dunning et al ^11^ reports a trend of increasing ratio of biological to mechanical AVR. Considering only the figures for patients aged <55 years old gives an estimated ratio of 0.3 for 2016, which implies a proportion biological of 0.23. To assess uncertainty on this figure, we needed the number of patients <55 years old of the 41,227 patients in the Dunning cohort. Assuming a similar proportion to that reported in the Society for Cardiothoracic Surgery in Great Britain & Ireland (SCTS) annual report ^12^, the number of patients is approximately 10,987, giving the distribution reported in Table S2.

***Cost and utility model inputs***

Several sources were used to estimate the state-occupancy and event costs and utilities (Table S2), including evidence from the extensive literature on stroke prevention in atrial fibrillation ^13-16^. The cost of the initial AVR and Ross was a combination of the 2016/17 NHS Tariff procedure costs and estimated implant costs from Brecker ^3, 17^. Costs of one-off events were also estimated according to the 2016/17 tariff. Cost estimates based on the NHS tariff were assumed fixed, as they represent the agreed prices paid to hospitals for activity. The age-dependent cost per year for an otherwise healthy patient (following AVR or Ross) was assumed to follow estimated NHS health care costs ^18^; published evidence was used to estimate the additional annual cost following a bleed or disabling stroke ^9, 13^, which were assumed the same following a published atrial fibrillation model ^15, 16^.

The healthy state utility followed that of Doble et al. but was adjusted downwards with age ^4, 19^. Utilities for stroke were adapted from Garcia et al. ^20^ and again following atrial fibrillation models, we assumed the same annual utilities for a history of bleed and non-disabling stroke ^15, 16^. In addition to utilities for each cycle, we associated disutilities with stroke, bleeds and the transient events (Table S1).

***Technology horizon and population size for EVPI and EVPPI***

A technology horizon for the EVPI refers to the timespan over which the research will influence practice. As the Ross procedure has been in use for 50 years, its only main competitor is AVR, and with no major breakthrough in growing personalised valves in vitro using stem cells, we adopted a 25-year technology horizon.

As the EVPI and EVPPI are per-person, we need an estimate of the discounted cumulative population eligible for AVR or Ross over a 10-year technology obsolescence time horizon to calculate population EVPI and EVPPI. The SCTS book reports 5796 isolated AVR operations in 2015, and extrapolating the increasing trend suggests 6000 in 2018 ^21^. To get the percentage of AVR that are 16-60, The Sixth National Adult Cardiac Surgical Database Report of 2008 indicates that there would be about 20% <61 years old by 2016 ^12^. Recent works suggests up to 80% of these would be eligible for the Ross procedure, giving 16% of 6000 and a total population eligible for Ross per year of 960 ^22^. Extrapolating to an average 960 patients annually eligible for Ross/AVR over the 25-year technology horizon and discounting at 3.5% per year gives a total population of 16376.

***Sensitivity analysis comparing Ross to bioAVR or mechAVR alone***

This sensitivity analysis assumes all AVR are biological or mechanical, rather than the 23/76 split of the base case. Results are presented in Table S2. Base case assumes a mean of 23% is biological while sensitivities set this to 0% (all mechAVR) or 100% (All bioAVR). Finding is that bioAVR is slightly worse (on both costs and quality of life outcomes) than mechAVR. We also found the EVPI to increase in both sensitivities. Potential explanation is that mechAVR is closer to Ross so decision becomes more uncertain and thus EVPI increases. For bioAVR, although it is further away from Ross on costs and QALYs the evidence is weaker (wider credible intervals) so the decision again becomes more uncertain and EVPI increases.

***Deterministic sensitivity analyses on 15 cost and utility parameters***

As some costs and utility parameter were fixed these would not be included in our primary sensitivity analyses of PSA and EVPI. These 15 parameters were costs of the Ross procedure, costs of AVR, cost of IE conservatively treated, cost of AoV reoperation for IE, cost of AoV reoperation for other causes, cost of stroke event, cost of bleeding event, cost of PV reoperation, cost of PV catheter-based procedure, cost of concomitant AoV and PV reoperation, disutility after AoV reoperation, disutility after PV reoperation, disutility after PV catheter-based procedure, disutility after concomitant AoV and PV reoperation, and cost of a non-disabling stroke or bleed. We conducted one-way sensitivity analyses where these parameters were set to 50% and 150% of their values.

**Table describing model input parameters, their assumptions and evidence sources**

| **Supplemental Table S1.** Input parameter values and sources used in the cost-effectiveness model | | | |
| --- | --- | --- | --- |
| **Parameter** | **Value** | **Source and comments** | |
| **Epidemiological parameters** | | | |
| Probability of death following stroke | Normal(mean=0.15, sd=0.025) | Weimar 2002 ^23^ as used in Garcia 2005 ^20^ | |
| Probability of death following IE conservatively treated | Normal(mean=0.25, sd=0.056) | Tornos 1997, discussed in Tornos 2003, reports 25% in 59 patients ^24, 25^. Uncertainty interval in line with with 21.5% rate reported in Luciani 2017 ^26^. | |
| Probability of death following (non-haemorrhagic stroke) bleed | 0.05 (0, 0.1) | Limited evidence on the mortality rates following a major bleeding event and under clinical guidance, we assumed mean probabilities 0.05 with a vague uniform distribution ^27, 28^ . | |
| Proportion of stroke that is disabling | Normal(mean=0.4, sd=0.10) | Weimar 2002 ^23^ as used in Garcia 2005 ^20^ | |
| Proportion AVR that is tissue/biological | Normal(mean=0.23, sd=0.004) | Ratio of 0.3 for 2016/17 extrapolated from ratios reported in Dunning et al ^11^ giving proportion 0.23. Number of patients for SE estimates as 10,987 assuming same proportion of Dunning cohort are <55 as in SCTS annual report ^12^ | |
| Log odds of death following IE AoV reop | Normal(mean=-1.672, SD=0.8796) | Random effects meta-analysis on log odds scale in WinBUGS of David 2009 [REF]^29^, Wang 2005 ^30^, Perrotta 2016 ^31^. Use inverse logistic transformation to provide probabilities. | |
| Probability of death following AoV reop | Normal(mean= 0.077, SD=0.015) | Onorati 2014 ^32^ | |
| Probability of death following PV reop | 0.01 | Assumption. Lee 2016 found 0 deaths in 61 PV reoperation patients ^33^ | |
| Probability of death following PV catheter procedure | 0 | Nordmeyer2009 found 0 deaths in 12 patients ^34^ | |
| Probability of stroke following IE AoV reop | Normal(mean=0.13, SD=0.036) | Perrotta 2016 ^31^ | |
| Probability of stroke following AoV reop | Normal(mean=0.071, SD=0.014) | Onorati 2014 ^32^ | |
| Probability of stroke following PV reop | Normal(mean=0.016, SD=0.016) | Lee 2016 ^33^ | |
| Probability of stroke following PV catheter procedure | 0 | Nordmeyer 2009 found 0 stroke in 12 patients ^34^ | |
| Probability of stroke and death following Concomitant AoV PV reop | Assumed same as for AoV reop |  | |
| Probability of Stroke following tissue AVR | 0.0543 (0.000495, 0.444) | Random effects meta-analysis of studies reporting short-term (<30 day) complications | |
| Probability of Bleed following tissue AVR | 0.0382 (7.42e-05, 0.362) |  |  |
| Probability of Stroke following mechanical AVR | 0.0246 (0.00221, 0.0927) |  |  |
| Probability of Bleed following mechanical AVR | 0.0454 (0.00393, 0.182) |  |  |
| Probability of Stroke following Ross | 0.00552 (0.00108, 0.0116) |  |  |
| Probability of Bleed following Ross | 0.0349 (0.0109, 0.0732) |  |  |
| **Results of random effects meta-analysis** | | | |
|  | **Tissue AVR, mean (95% CI)** | **Mechanical AVR, mean (95% CI)** | **Ross, mean (95% CI)** |
| Bleed | 0.0031 (0.0014- 0.0069) | 0.0069 (0.0050- 0.0096) | 0.0011 (0.0006- 0.0020) |
| Death | 0.0254 (0.0180- 0.0359) | 0.0138 (0.0111- 0.0174) | 0.0054 (0.0045- 0.0064) |
| Concomitant AoV PV reop | Assumed 0 | Assumed 0 | 0.0013 (0.0010- 0.0017) |
| IE cons treat | 0.0029 (0.0010- 0.0087) | 0.0027 (0.0018- 0.0042) | 0.0021 (0.0015- 0.0030) |
| Stroke | 0.0057 (0.0039- 0.0084) | 0.0086 (0.0062- 0.0118) | 0.0026 (0.0019- 0.0037) |
| AoV reop | 0.0128 (0.0088- 0.0185) | 0.0037 (0.0027- 0.0050) | 0.0054 (0.0042- 0.0069) |
| PV reop | Assumed 0 | Assumed 0 | 0.0043 (0.0034- 0.0054) |
| PV catheter | Assumed 0 | Assumed 0 | 0.0015 (0.0008- 0.0026) |
| IE AoV reop | 0.0026 (0.0018- 0.0039) | 0.0017 (0.0010- 0.0027) | 0.0016 (0.0012- 0.0020) |
| **Cost parameters** | | | |
| AVR procedure and implant cost | £10900.52 | Assumed same for tissue and mechanical. Used implant cost reported in Brecker (£2126.52) plus 2016/17 Tariff (HRG EA17Z Single Cardiac Valve Procedures); includes stays up to 17 days, elective procedure ^3^ [REF Tariff] | |
| Ross procedure and implant cost | £12707.52 | Becker implant cost (£2126.52) [REF] plus 2016/17 Tariff (HRG EA52Z Repair or Replacement of more than one Heart Valve); includes stays up to 33 days; elective procedure ^3^ [REF Tariff] | |
| Cost per year for otherwise healthy patient | Depends on patient age | Centre for health economics research paper 147, health care costs in NHS ^18^ | |
| Additional cost per year for post bleed | £740.62 | Fairbairn anticoagulant treatment post-bleed £649.00, from BNF, inflated to 2016/17 prices ^9^. | |
| Additional cost per year for post stroke no disability | Assumed same as post bleed (£740.62) |  | |
| Additional cost per year of stroke disability | Normal(mean=515, SD=874) | Weighted average of Luengo 2012 moderately and totally disabling costs (post - pre), inflated from 2009 to 2016/17 prices ^13^ | |
| Bleed event cost | £2347.611 | Weighted average of 2016/17 Tariffs HRG FZ38D (Gastrointestinal Bleed, with length of stay 2 days or more, with Major CC) and HRG FZ38E (Gastrointestinal Bleed, with length of stay 2 days or more, without Major CC) ^17^ | |
| Stroke event cost | £4375 | 2016/17 Tariff (AA04B Major Intracranial Procedures Except Trauma with Non-Transient Stroke or Cerebrovascular Accident, Nervous System Infections or Encephalopathy, without CC) ^17^ | |
| Infective endocarditis, Conservative treatment, event cost | £2555 | 2016/17 Tariff (EB02Z Endocarditis) | |
| Infective endocarditis requiring AV V Reoperation | £10,821.00 | 2016/17 Tariff (HRG EA17Z Single Cardiac Valve Procedures); includes stays up to 55 days, non-elective procedure | |
| Aortic valve reoperation | £10,821.00 | 2016/17 Tariff (HRG EA17Z Single Cardiac Valve Procedures); includes stays up to 55 days, non-elective procedure | |
| PV reoperation | £10,821.00 | 2016/17 Tariff (HRG EA17Z Single Cardiac Valve Procedures); includes stays up to 55 days, non-elective procedure | |
| PV catheter procedure | £15,736.24 | Device cost from Brecker ^3^ inflated from 2013/14 to 2016/17 prices, plus procedure cost 2016/17 Tariff (EA53Z Transcatheter Aortic Valve Implantation (TAVI)); includes stays up to 55 days, non-elective procedure | |
| Concomitant AV and PV reintervention | £14,009.00 | 2016/17 Tariff (HRG EA52Z Repair or Replacement of more than one Heart Valve); includes stays up to 71 days; non-elective procedure | |
| **Utility and quality of life parameters** | | | |
| Disutility for AoV reoperation | -0.1 | 10% disutility for "After aortic valve replacement" used in Gada 2012 ^8^ | |
| Disutility for PV reoperation | -0.1 |  |  |
| Disutility for PV catheter procedure | -0.1 |  |  |
| Disutility for Concomitant AoV and PV reoperation | -0.1 |  |  |
| Disutility for Infective endocarditis, Conservative treatment. | Beta(4, 0.5)-Beta(56, 0.69) (mean=-0.1, SD=0.13) | Following Doble 2013 ^4^. Rosen 1999 gives Alive without complications while Heidenreich 1999 gives IE ^35, 36^. Difference is disutility (for one cycle) for IE. Randomly sampled from beta distributions but capped above at zero to ensure negative. Assumed same for conservatively and surgically treated. | |
| Disutility for Infective endocarditis, AoV reoperation. |  |  |  |
| Disutility for bleed event | Normal(mean=-0.03, sd=0.0015) | Atrial fibrillation model reports disutility for "Other clinically relevant bleeding (not including haemorrhagic stroke) disutility ^15, 16^, based on Robinson 2001 ^14^. | |
| Disutility for stroke (non-disabling or disabling) | Uniform(-0.885, -0.295) | As in atrial fibrillation model, based on Robinson 2001 ^14-16^. | |
| Healthy state utility per cycle | Beta(56, 0.69) (mean=0.988, sd=0.014) | Based on Doble 2013 Table E1 ^4^ for Alive without complications, source Rosen 1999 ^36^. This is scaled down with age using Kind et al factors ^19^. | |
| Stroke no disability state utility per cycle | Normal(mean=0.75, sd=0.077) | Garcia 2005 range for "Stroke with minor deficit" ^20^ | |
| Stroke disability state utility per cycle | Normal(mean=0.5, sd=0.153) | Garcia 2005 range for "Stroke with major deficit" ^20^ | |
| Bleed state utility per cycle | Normal(mean=0.75, sd=0.077) | Assumed same as non-disabling stroke, following published atrial fibrillation model ^15, 16^ | |

| **Supplemental Table S2.** Sensitivity analysis comparing Ross to mechAVR and bioAVR | | | | | | |
| --- | --- | --- | --- | --- | --- | --- |
|  | **Base case (mean prop bio 23%)** | | **Prop bio 0% (all mechanical)** | | **Prop bio 100% (all biological)** | |
|  | **SVR** | **Ross** | **SVR** | **Ross** | **SVR** | **Ross** |
| **Costs** | 33820 (31024, 39865) | 46127 (44276, 48224) | 35174 (32124, 41844) | 46132 (44262, 48261) | 31023 (26643, 45603) | 46119 (44283, 48237) |
| **QALYs** | 11.5 (10.4, 13.3) | 15.2 (14.4, 15.9) | 12 (10.9, 14) | 15.2 (14.3, 15.9) | 10.3 (8.7, 14.6) | 15.2 (14.4, 15.9) |
| **NB at £20,000** | 196405 (177178, 226878) | 257311 (241876, 270800) | 205137 (184736, 238024) | 257276 (241594, 270598) | 174972 (146964, 247651) | 257224 (241743, 270585) |
| **NB at £50,000** | 541743 (490260, 626748) | 712468 (672674, 747958) | 565604 (510826, 658605) | 712389 (672214, 747934) | 483964 (408027, 686394) | 712239 (672504, 747733) |
| **Incremental costs** | 0 (0, 0) | 12306 (6066, 15933) | 0 (0, 0) | 10958 (4124, 14795) | 0 (0, 0) | 15096 (517, 20172) |
| **Incremental QALYs** | 0 (0, 0) | 3.66 (1.83, 4.76) | 0 (0, 0) | 3.15 (1.22, 4.32) | 0 (0, 0) | 4.87 (0.559, 6.56) |
| **Incremental NB at £20,000** | 0 (0, 0) | 60906 (30340, 79509) | 0 (0, 0) | 52139 (19884, 71970) | 0 (0, 0) | 82252 (11396, 111457) |
| **Incremental NB at £50,000** | 0 (0, 0) | 170725 (85329, 222170) | 0 (0, 0) | 146784 (56375, 201614) | 0 (0, 0) | 228275 (28579, 308123) |
| **EVPI** | 123.7 (118, 129.4) | | 476.8676 | | 501.993 | |
| **EVPI pop** | 2,026,008 (1,932,365; 2,119,651) | | 3805081 | | 4005566 | |

***SUPPLEMENTARY MATERIAL B REFERENCES***

1. Briggs AH, Claxton K, Sculpher MJ. *Decision modelling for health economic evaluation*. Oxford: Oxford University Press; 2006.

2. Siebert U, Alagoz O, Bayoumi AM, Jahn B, Owens DK, Cohen DJ, Kuntz KM. State-Transition Modelling: A report of the ISPOR-SMDM Modelling Good Research Practices Task Force-3. Value in Health 2012;**15**:812-820.

3. Brecker S, Mealing S, Padhiar A, Eaton J, Sculpher M, Busca R, Bosmans J, Gerckens UJ, Wenaweser P, Tamburino C, Bleiziffer S, Piazza N, Moat N, Linke A. Cost-utility of transcatheter aortic valve implantation for inoperable patients with severe aortic stenosis treated by medical management: a UK cost-utility analysis based on patient-level data from the ADVANCE study. Open Heart 2014;**1**(1):e000155.

4. Doble B, Blackhouse G, Goeree R, Xie F. Cost-effectiveness of the Edwards SAPIEN transcatheter heart valve compared with standard management and surgical aortic valve replacement in patients with severe symptomatic aortic stenosis: a Canadian perspective. J Thorac Cardiovasc Surg 2013;**146**(1):52-60 e3.

5. Fairbairn TA, Meads DM, Hulme C, Mather AN, Plein S, Blackman DJ, Greenwood JP. The cost-effectiveness of transcatheter aortic valve implantation versus surgical aortic valve replacement in patients with severe aortic stenosis at high operative risk. Heart 2013;**99**(13):914-20.

6. Murphy A, Fenwick E, Toff WD, Neilson MP, Berry C, Uren N, Oldroyd KG, Briggs AH. Transcatheter aortic valve implantation for severe aortic stenosis: the cost-effectiveness case for inoperable patients in the United Kingdom. Int J Technol Assess Health Care 2013;**29**(1):12-9.

7. Watt M, Mealing S, Eaton J, Piazza N, Moat N, Brasseur P, Palmer S, Busca R, Sculpher M. Cost-effectiveness of transcatheter aortic valve replacement in patients ineligible for conventional aortic valve replacement. Heart 2012;**98**(5):370-6.

8. Gada H, Kapadia SR, Tuzcu EM, Svensson LG, Marwick TH. Markov model for selection of aortic valve replacement versus transcatheter aortic valve implantation (without replacement) in high-risk patients. Am J Cardiol 2012;**109**(9):1326-33.

9. Fairbairn TA, Meads DM, Mather AN, Motwani M, Pavitt S, Plein S, Blackman DJ, Greenwood JP. Serial change in health-related quality of life over 1 year after transcatheter aortic valve implantation: predictors of health outcomes. J Am Coll Cardiol 2012;**59**(19):1672-80.

10. Reynolds MR, Lei Y, Wang K, Chinnakondepalli K, Vilain KA, Magnuson EA, Galper BZ, Meduri CU, Arnold SV, Baron SJ, Reardon MJ, Adams DH, Popma JJ, Cohen DJ, CoreValve USHRPTI. Cost-Effectiveness of Transcatheter Aortic Valve Replacement With a Self-Expanding Prosthesis Versus Surgical Aortic Valve Replacement. J Am Coll Cardiol 2016;**67**(1):29-38.

11. Dunning J, Gao H, Chambers J, Moat N, Murphy G, Pagano D, Ray S, Roxburgh J, Bridgewater B. Aortic valve surgery: marked increases in volume and significant decreases in mechanical valve use--an analysis of 41,227 patients over 5 years from the Society for Cardiothoracic Surgery in Great Britain and Ireland National database. J Thorac Cardiovasc Surg 2011;**142**(4):776-782 e3.

12. Bridgewater B, Keogh B, Kinsman R, Walton P. Sixth National Adult Cardiac Surgical Database Report 2008: Demonstrating quality. In. July 2009 ed: Society for Cardiothoracic Surgery in Great Britain & Ireland; 2009.

13. Luengo-Fernandez R, Yiin GS, Gray AM, Rothwell PM. Population-based study of acute- and long-term care costs after stroke in patients with AF. Int J Stroke 2013;**8**(5):308-14.

14. Robinson A, Thomson R, Parkin D, Sudlow M, Eccles M. How patients with atrial fibrillation value different health outcomes: a standard gamble study. J Health Serv Res Policy 2001;**6**(2):92-8.

15. Sterne J, Bodalia P, Caldwell D, Hingorani A, Hollingworth W, Salisbury C, Welton N, Savovic J, Higgins J, Casas JP. HTA - 11/92/17: Oral anticoagulants for primary prevention, treatment and secondary prevention of venous thromboembolic disease, and for prevention of stroke in atrial fibrillation: systematic review, network meta-analysis and cost-effectiveness NIHR HTA 2017.

16. Lopez-Lopez JA, Sterne J, Thom H, Higgins J, Hingorani A, Okoli GN, Davies PA, Bodalia P, Bryden PA, Welton N, Hollingworth W, Caldwell D, Savovic J, Dias S, Salisbury C, Eaton D, Stephens-Boal A, Sofat R. Oral anticoagulants for prevention of stroke in atrial fibrillation: systematic review, network meta-analysis, and cost effectiveness analysis. BMJ 2017;**359**(J5058).

17. Monitor. 2016/17 National Tariff Payment System. <https://www.gov.uk/government/publications/nhs-national-tariff-payment-system-201617>. Accessed online 8th Feb 2018. 2016.

18. Asaria M. *Health care costs in the English NHS: reference tables for average annual NHS spend by age, sex and deprivation group. CHE Research Paper 147.* [*https://www.york.ac.uk/media/che/documents/papers/researchpapers/CHERP147_health_care_costs_NHS.pdf*:](https://www.york.ac.uk/media/che/documents/papers/researchpapers/CHERP147_health_care_costs_NHS.pdf:) York Centre for Health Economics; 2017.

19. Kind P, Hardman G, Macran S, University of York. Centre for Health Economics. *UK population norms for EQ-5D*. York: University of York, Centre for Health Economics; 1999.

20. Garcia D, Libby E, Rich S. Perioperative anticoagulation for patients with mechanical heart valves: A model comparing unfractionated and low-molecular-weigth heparin. Journal of Clinical Outcomes Management 2005;**12**(1).

21. The Society for Cariothoracic Surgery in Great Britain & Ireland. Blue Book Online (<http://bluebook.scts.org/>). In. United Kingdom: Healthcare Quality Improvement Partnership and National Institute for Cardiovascular Outcomes Research; 2016.

22. Ghoneim A, Bouhout I, Losenno K, Poirier N, Cartier R, Demers P, Tousch M, Linruo G, Chu MWA, El-Hamamsy I. Expanding Eligibility for the Ross Procedure: A Reasonable Proposition. Can J Cardiol 2018.

23. Weimar C, Kurth T, Kraywinkel K, Wagner M, Busse O, Haberl RL, Diener HC, German Stroke Data Bank C. Assessment of functioning and disability after ischemic stroke. Stroke 2002;**33**(8):2053-9.

24. Tornos P, Almirante B, Olona M, Permanyer G, Gonzalez T, Carballo J, Pahissa A, Soler-Soler J. Clinical outcome and long-term prognosis of late prosthetic valve endocarditis: a 20-year experience. Clin Infect Dis 1997;**24**(3):381-6.

25. Tornos P. Management of prosthetic valve endocarditis: a clinical challenge. Heart 2003;**89**(3):245-6.

26. Luciani N, Mossuto E, Ricci D, Luciani M, Russo M, Salsano A, Pozzoli A, Pierri MD, D'Onofrio A, Chiariello GA, Glieca F, Canziani A, Rinaldi M, Nardi P, Milazzo V, Trecarichi EM, Santini F, De Bonis M, Torracca L, Bizzotto E, Tumbarello M. Prosthetic valve endocarditis: predictors of early outcome of surgical therapy. A multicentric study. Eur J Cardiothorac Surg 2017;**52**(4):768-774.

27. Skaistis J, Tagami T. Risk of Fatal Bleeding in Episodes of Major Bleeding with New Oral Anticoagulants and Vitamin K Antagonists: A Systematic Review and Meta-Analysis. PLoS One 2015;**10**(9):e0137444.

28. Conti A, Renzi N, Molesti D, Bianchi S, Bogazzi I, Bongini G, Pepe G, Frosini F, Bertini A, Santini M. Short and long-term mortality of patients presenting with bleeding events to the Emergency Department. Am J Emerg Med 2017;**35**(12):1867-1872.

29. David TE, Gavra G, Feindel CM, Regesta T, Armstrong S, Maganti MD. Surgical treatment of active infective endocarditis: a continued challenge. J Thorac Cardiovasc Surg 2007;**133**(1):144-9.

30. Wang A, Pappas P, Anstrom KJ, Abrutyn E, Fowler VG, Jr., Hoen B, Miro JM, Corey GR, Olaison L, Stafford JA, Mestres CA, Cabell CH, International Collaboration on Endocarditis I. The use and effect of surgical therapy for prosthetic valve infective endocarditis: a propensity analysis of a multicenter, international cohort. Am Heart J 2005;**150**(5):1086-91.

31. Perrotta S, Jeppsson A, Frojd V, Svensson G. Surgical Treatment of Aortic Prosthetic Valve Endocarditis: A 20-Year Single-Center Experience. Ann Thorac Surg 2016;**101**(4):1426-32.

32. Onorati F, Biancari F, De Feo M, Mariscalco G, Messina A, Santarpino G, Santini F, Beghi C, Nappi G, Troise G, Fischlein T, Passerone G, Heikkinen J, Faggian G. Mid-term results of aortic valve surgery in redo scenarios in the current practice: results from the multicentre European RECORD (REdo Cardiac Operation Research Database) initiativedagger. Eur J Cardiothorac Surg 2015;**47**(2):269-80; discussion 280.

33. Lee C, Lee CH, Kwak JG. Outcomes of redo pulmonary valve replacement for bioprosthetic pulmonary valve failure in 61 patients with congenital heart disease. Eur J Cardiothorac Surg 2016;**50**(3):470-5.

34. Nordmeyer J, Lurz P, Tsang VT, Coats L, Walker F, Taylor AM, Khambadkone S, de Leval MR, Bonhoeffer P. Effective transcatheter valve implantation after pulmonary homograft failure: a new perspective on the Ross operation. J Thorac Cardiovasc Surg 2009;**138**(1):84-8.

35. Heidenreich PA, Masoudi FA, Maini B, Chou TM, Foster E, Schiller NB, Owens DK. Echocardiography in patients with suspected endocarditis: a cost-effectiveness analysis. Am J Med 1999;**107**(3):198-208.

36. Rosen AB, Fowler VG, Jr., Corey GR, Downs SM, Biddle AK, Li J, Jollis JG. Cost-effectiveness of transesophageal echocardiography to determine the duration of therapy for intravascular catheter-associated Staphylococcus aureus bacteremia. Ann Intern Med 1999;**130**(10):810-20.

***SUPPLEMENTAL MATERIAL C – META-ANALYSIS***

| **Supplementary Figure S3.** PRISMA flowchart for systematic literature review*    * PRISMA Flowchart: 48 isolated cohorts pulled from 43 studies resulted from a 2-stage screening of 8,370 initial articles. |
| --- |

1. ***SEARCH PROTOCOL FOR SYSTEMATIC LITERATURE REVIEW***

**PubMED / Medline (*4380 results*):**

("Heart Valve Prosthesis Implantation"[Mesh] OR (Heart[TiAb] AND Valve[TiAb] AND Insertion[TiAb]) OR (aortic[TiAb] AND valve[TiAb] AND replacement[TiAb])) AND (ross[TiAb] OR (pulmonary[TiAb] AND autograft[TiAb]) OR (mechanical[TiAb] AND valve[TiAb]) OR autograft[TiAb] OR (tissue[TiAb] AND valve[TiAb]) OR (biological[TiAb] AND valve[TiAb]) OR (biological[TiAb] AND prosthe*[TiAb]) OR bioprosthe*[TiAb] OR xenograft[TiAb] OR xenoprosthe*[TiAb] OR porcine[TiAb] OR bovine[TiAb]) AND (("1990/01/01"[PDAT] : "3000/12/31"[PDAT]) AND English[lang] AND ("adult"[MeSH] OR "adult"[MeSH Terms] OR "adolescent"[MeSH Terms] OR adult* OR (young adult*) OR (middle age*) OR (middle-age*)))

**PubMED / Medline – without age filter (*1447 extra results)*:**

(“Heart Valve Prosthesis Implantation”[Mesh] OR (Heart[TiAb] AND Valve[TiAb] AND Insertion[TiAb]) OR (aortic[TiAb] AND valve[TiAb] AND replacement[TiAb])) AND (ross[TiAb] OR (pulmonary[TiAb] AND autograft[TiAb]) OR (mechanical[TiAb] AND valve[TiAb]) OR autograft[TiAb] OR (tissue[TiAb] AND valve[TiAb]) OR (biological[TiAb] AND valve[TiAb]) OR (biological[TiAb] AND prosthe*[TiAb]) OR bioprosthe*[TiAb] OR xenograft[TiAb] OR xenoprosthe*[TiAb] OR porcine[TiAb] OR bovine[TiAb]) AND ((“1990/01/01”[PDAT] : “3000/12/31”[PDAT]) AND English[lang])

**EMBASE (*4104 results)*:**

(exp HEART VALVE REPLACEMENT/ OR heart valve implantation.mp. OR aortic valve replacement.mp. OR heart valve insertion.mp. OR exp AORTA VALVE REPLACEMENT/) AND (exp ROSS PROCEDURE/ OR pulmonary autograft.mp. OR exp HEART VALVE PROSTHESIS/ OR mechanical valve.mp. OR AUTOGRAFT/ OR tissue valve.mp. OR biological valve.mp. OR BIOPROSTHESIS/ OR XENOGRAFT/ OR porcine.mp.) limit to (english language and embase and yr="1990 -Current" and (adolescent <13 to 17 years> or adult <18 to 64 years>))

| 1. ***META-ANALYSIS FOREST PLOTS***   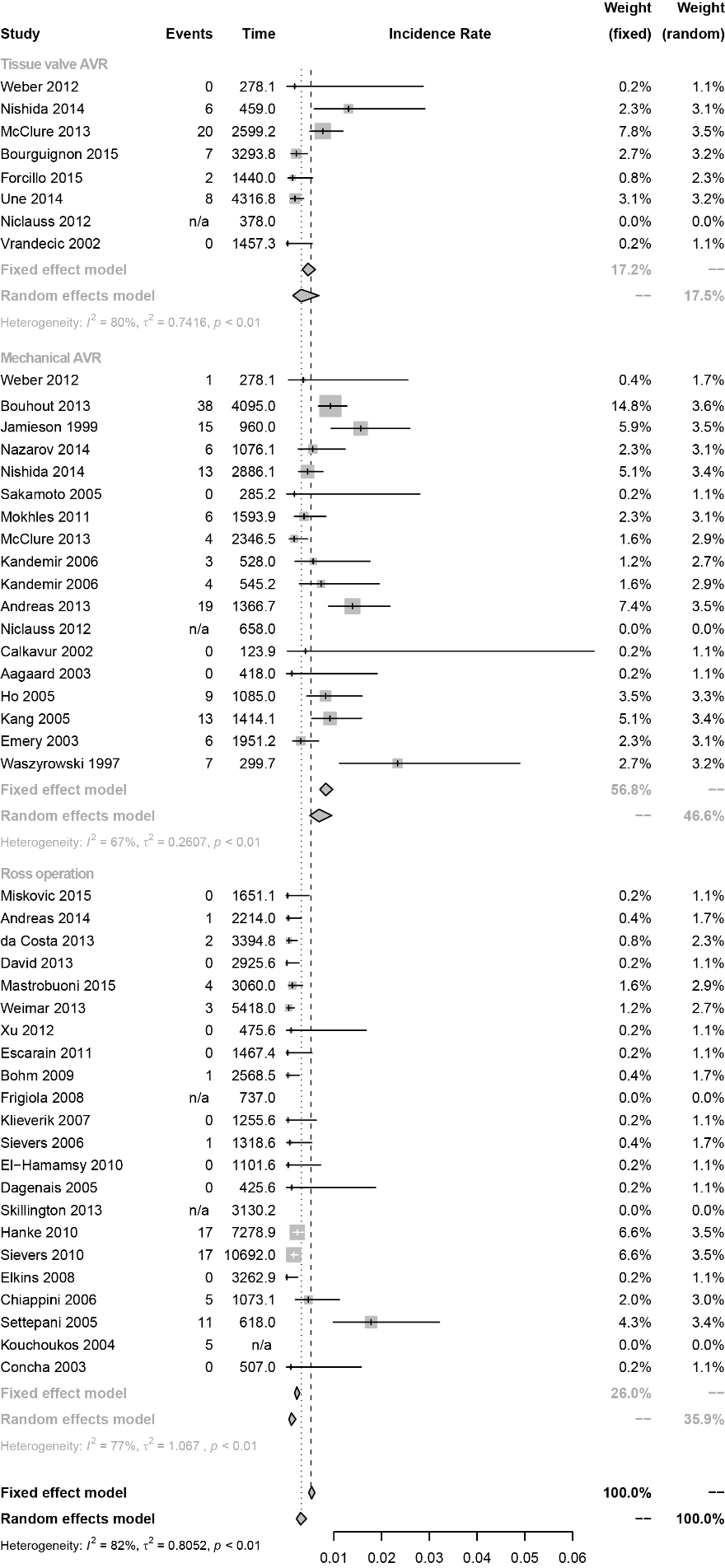 |
| --- |
| **Supplemental figure S4.** Early mortality forest plot. Ross procedure versus AVR with a tissue or mechanical prosthesis. Vertical marks = point estimate of RR; Horizontal lines = 95% CI; Solid squares = weight (fixed); Solid diamonds = estimated 95% CI of the meta-analysis (fixed and random). |
| 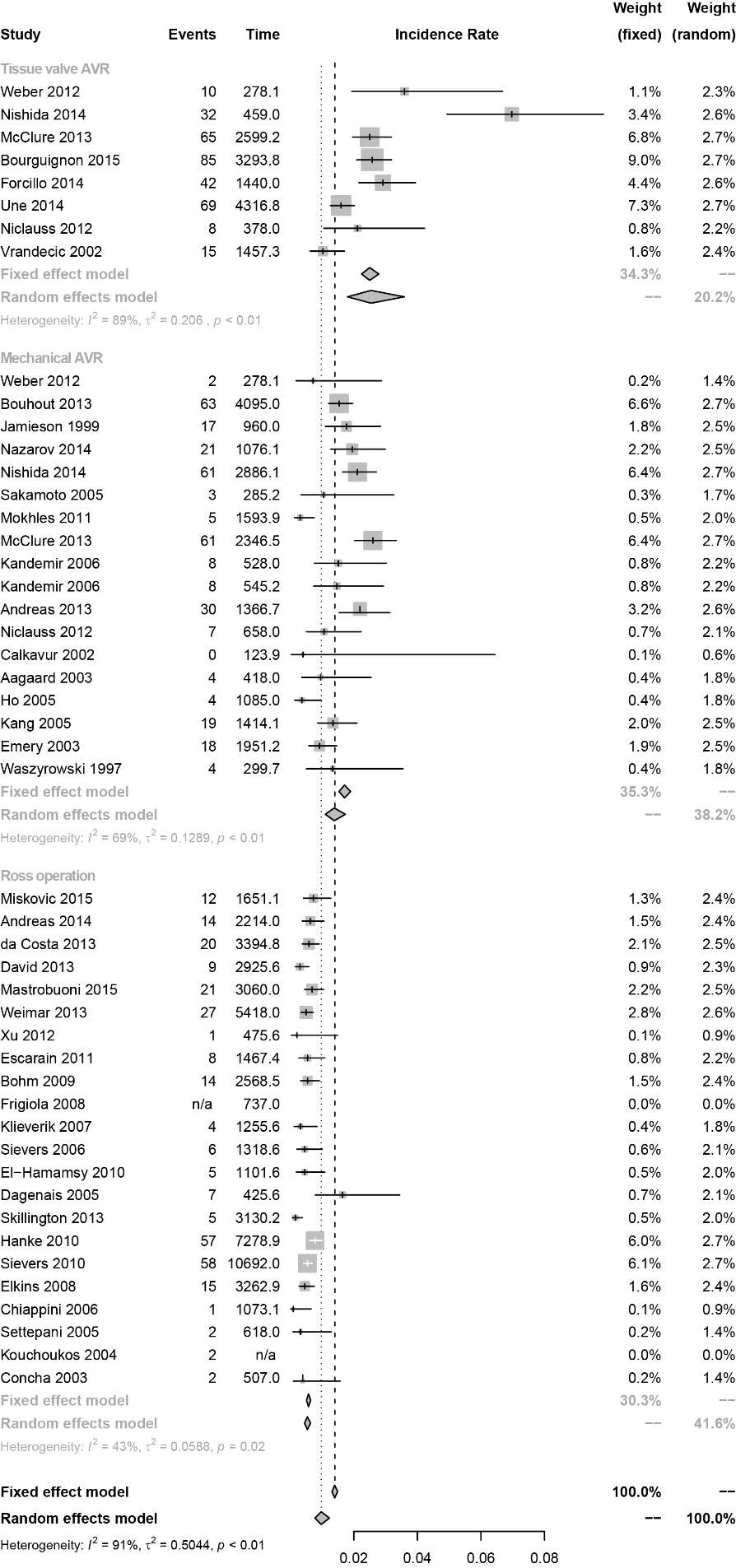 |
| **Supplemental figure S5.** Late mortality forest plot. Ross procedure versus AVR with a tissue or mechanical prosthesis. Vertical marks = point estimate of RR; Horizontal lines = 95% CI; Solid squares = weight (fixed); Solid diamonds = estimated 95% CI of the meta-analysis (fixed and random). |
| 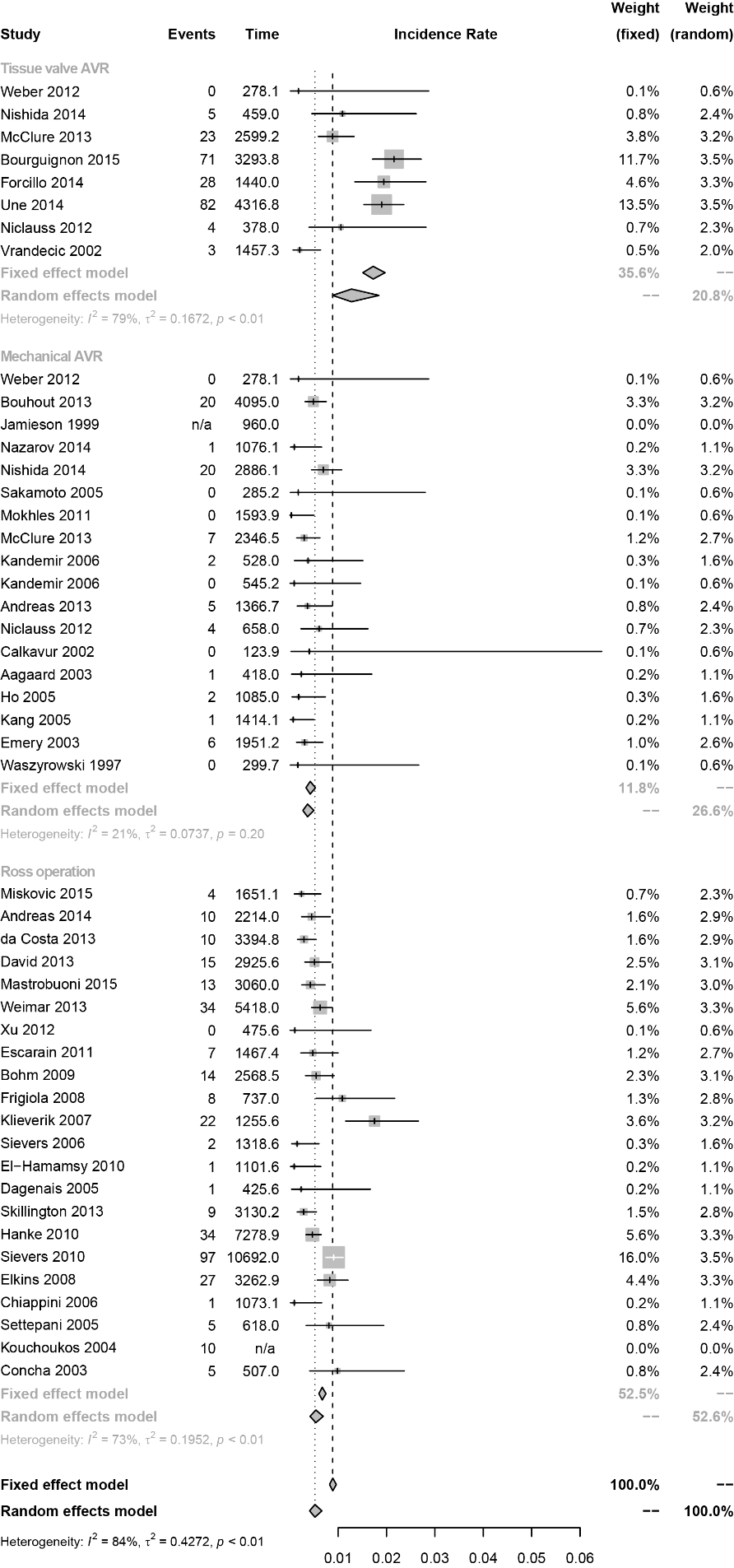 |
| **Supplemental figure S6.** Aortic valve reoperation forest plot. Ross procedure versus AVR with a tissue or mechanical prosthesis. Vertical marks = point estimate of RR; Horizontal lines = 95% CI; Solid squares = weight (fixed); Solid diamonds = estimated 95% CI of the meta-analysis (fixed and random). |
| 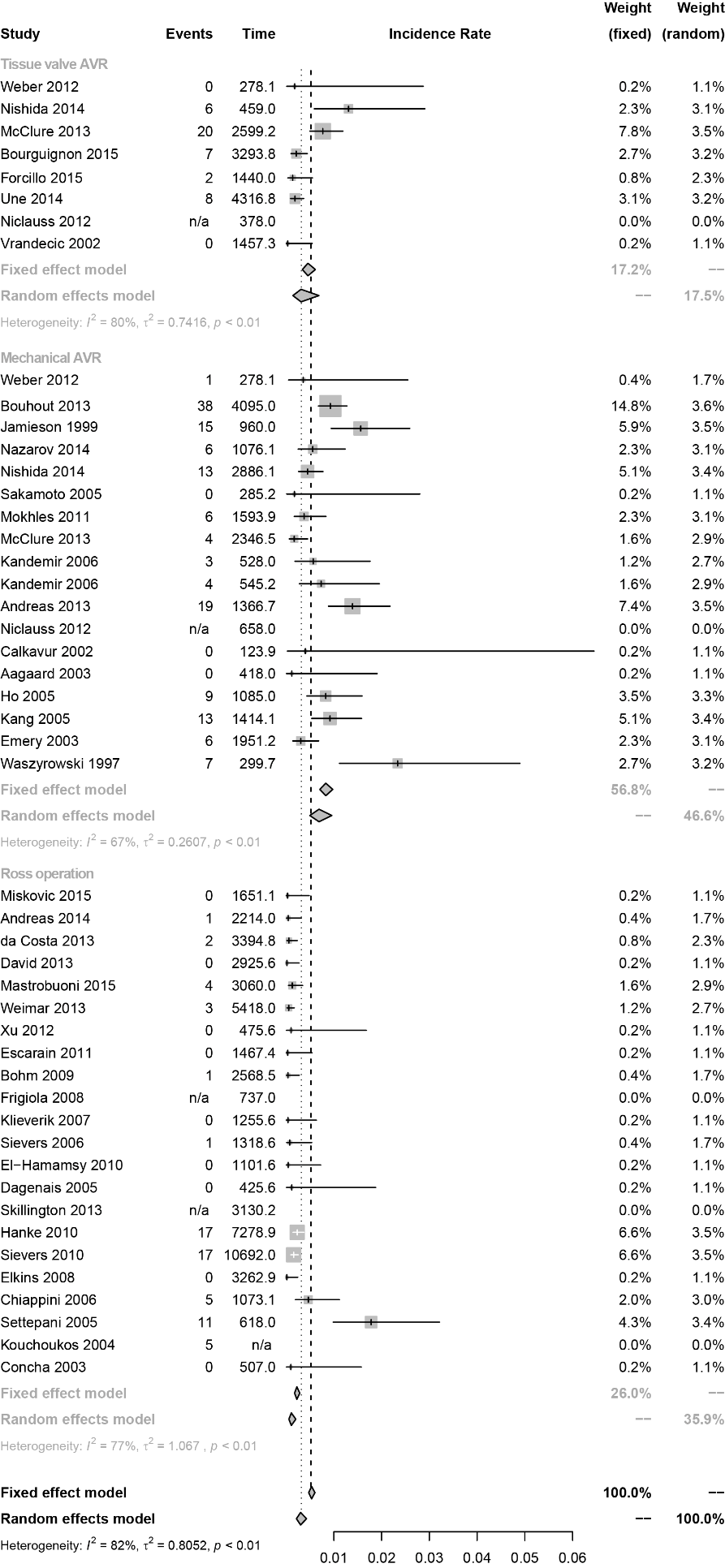 |
| **Supplemental figure S7.** Bleeding events forest plot. Ross procedure versus AVR with a tissue or mechanical prosthesis. Vertical marks = point estimate of RR; Horizontal lines = 95% CI; Solid squares = weight (fixed); Solid diamonds = estimated 95% CI of the meta-analysis (fixed and random). |
| 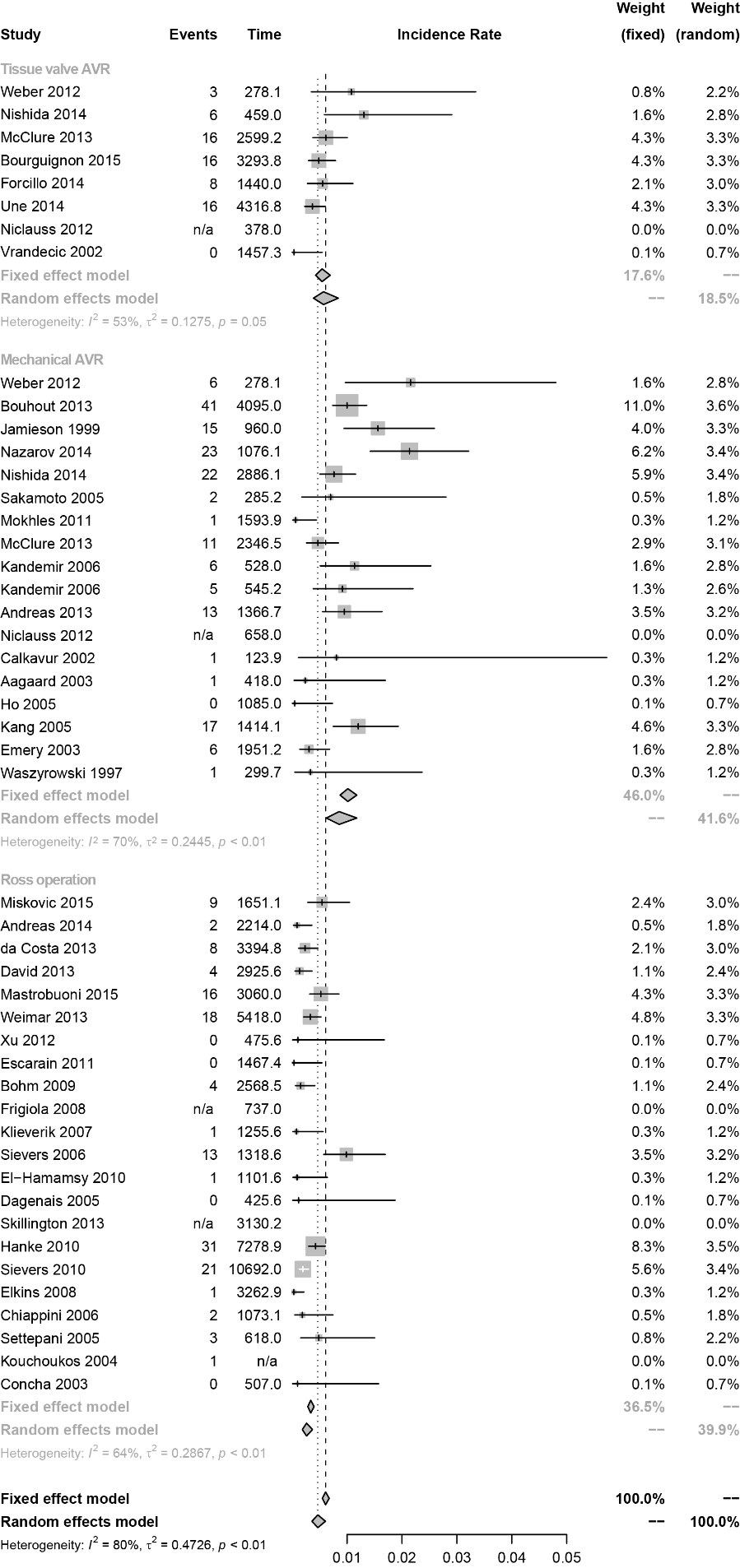 |
| **Supplemental figure S8.** Thrombembolic events forest plot. Ross procedure versus AVR with a tissue or mechanical prosthesis. Vertical marks = point estimate of RR; Horizontal lines = 95% CI; Solid squares = weight (fixed); Solid diamonds = estimated 95% CI of the meta-analysis (fixed and random). |
| 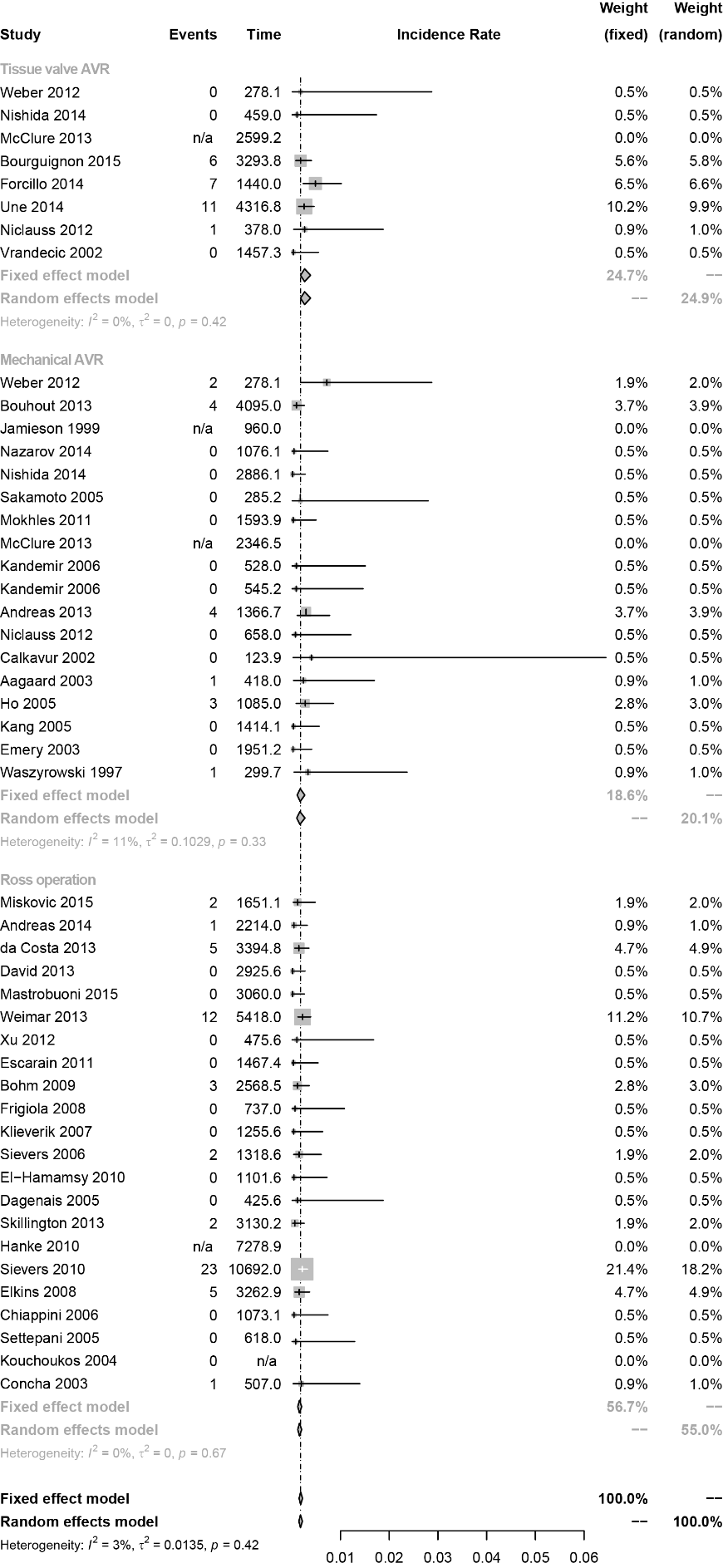 |
| **Supplemental figure S9.** Aortic valve reoperation for infective endocarditis forest plot. Ross procedure versus AVR with a tissue or mechanical prosthesis. Vertical marks = point estimate of RR; Horizontal lines = 95% CI; Solid squares = weight (fixed); Solid diamonds = estimated 95% CI of the meta-analysis (fixed and random). |
| 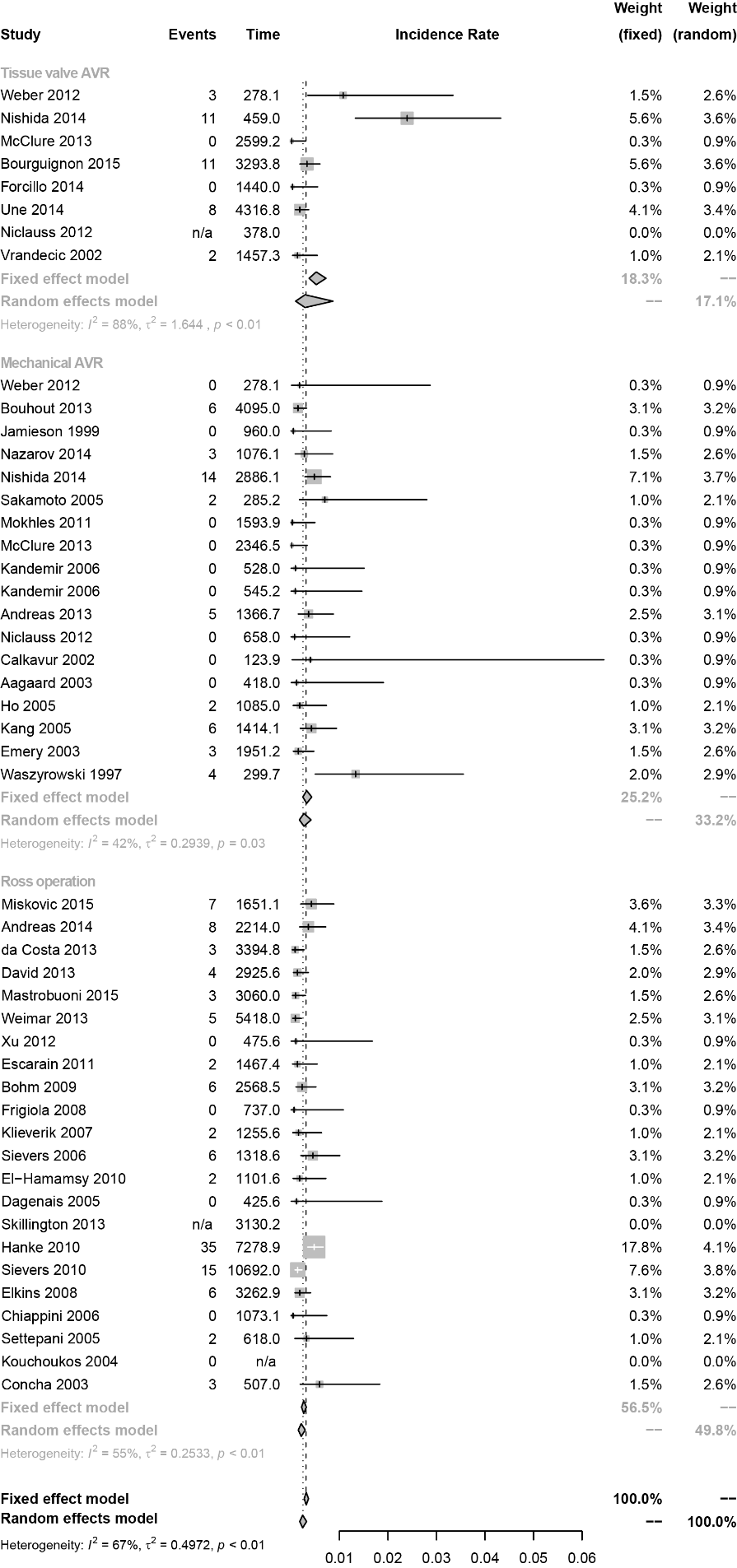 |
| **Supplemental figure S10.** Infective endocarditis treated conservatively. Ross procedure versus AVR with a tissue or mechanical prosthesis. Vertical marks = point estimate of RR; Horizontal lines = 95% CI; Solid squares = weight (fixed); Solid diamonds = estimated 95% CI of the meta-analysis (fixed and random). |
| 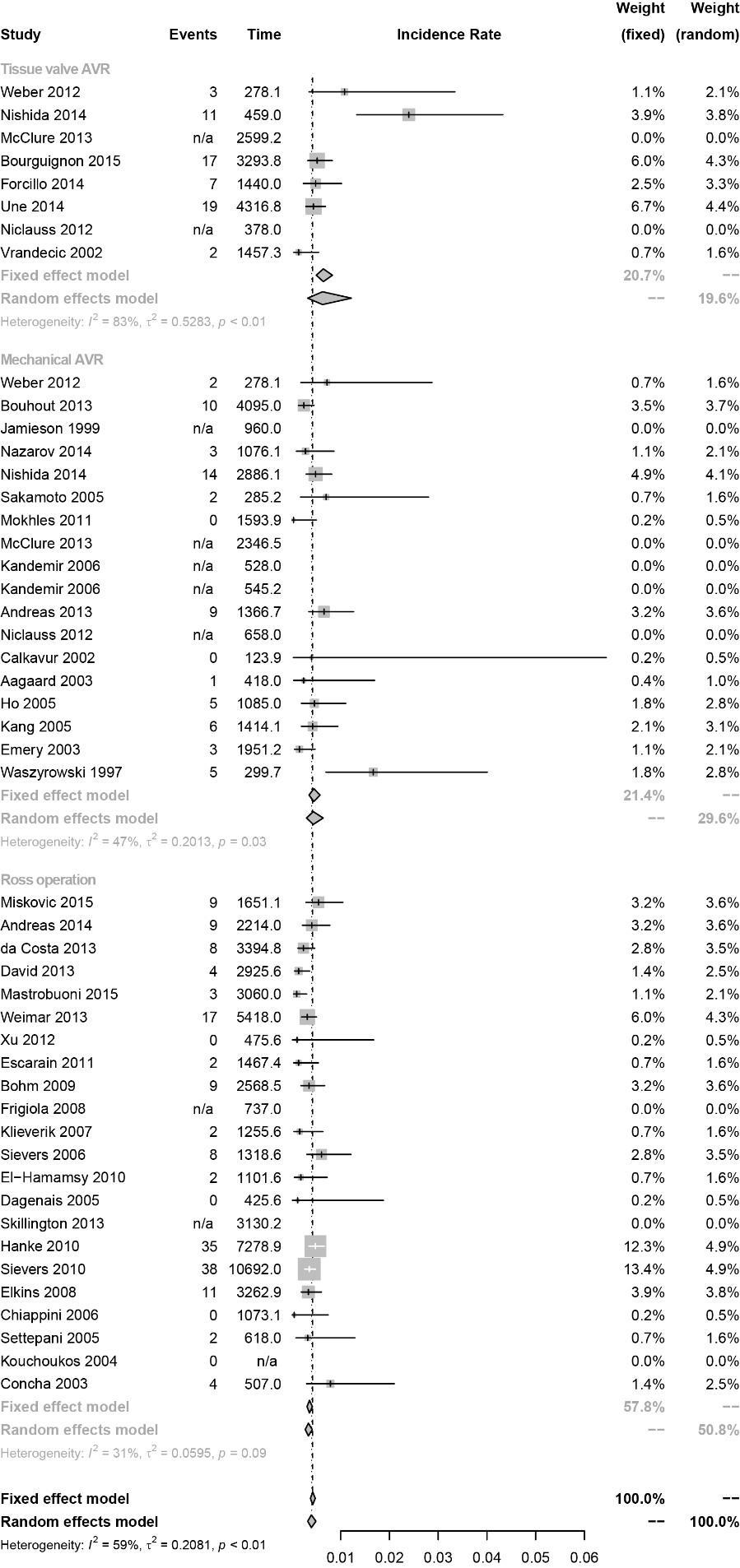 |
| **Supplemental figure S11.** Total infective endocarditis. Ross procedure versus AVR with a tissue or  mechanical prosthesis. Vertical marks = point estimate of RR; Horizontal lines = 95% CI; Solid squares  = weight (fixed); Solid diamonds = estimated 95% CI of the meta-analysis (fixed and random). |

| 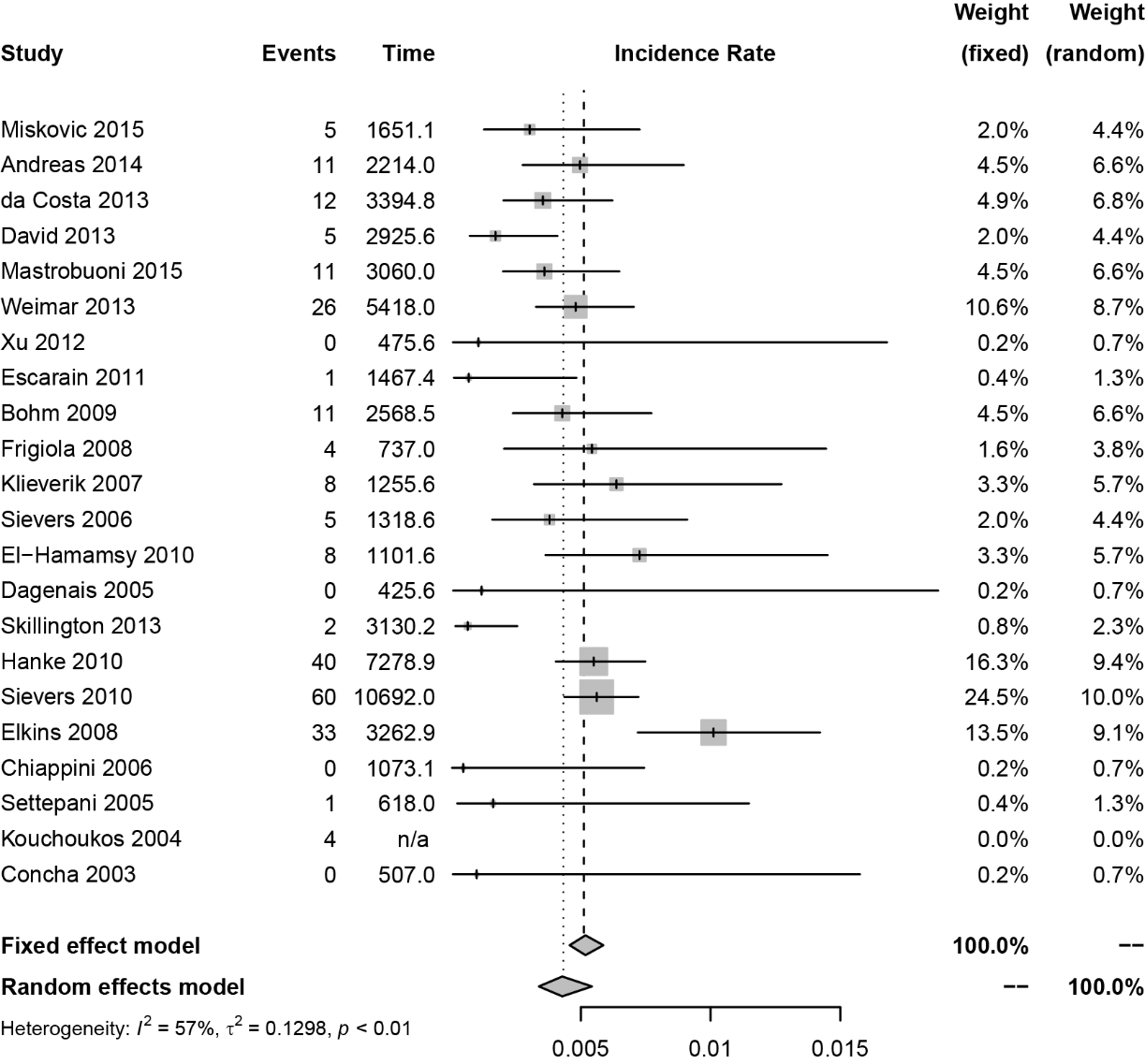 |
| --- |
| **Supplemental figure S12.** Right ventricular outflow tract reinterventions (Ross only). Vertical marks = point estimate of RR; Horizontal lines = 95% CI; Solid squares = weight (fixed); Solid diamonds = estimated 95% CI of the meta-analysis (fixed and random). |

1. ***META-ANALYSIS TABLES***

| Supplemental Table S3. Summary of publications used in the clinical meta-analysis. | | | | | |
| --- | --- | --- | --- | --- | --- |
| Author | Year | Patients | Age | Follow-up | Study type |
| **Biological** | | | | | |
| Weber ^1^ | 2012 | 103 | 55 | 2.7 | Retrospective |
| Nishida ^2^ | 2014 | 51 | 46.9 | 9.0 | Retrospective |
| McClure ^3^ | 2014 | 361 | 53.9 | 7.2 | Retrospective |
| Bourguignon ^4^ | 2015 | 383 | 51 | 8.6 | Retrospective |
| Forcillo ^5^ | 2014 | 144 | 51 | 10.0 | Retrospective |
| Une ^6^ | 2014 | 304 | 49.2 | 14.2 | Retrospective |
| Niclauss ^7^ | 2013 | 84 | 54.7 | 4.5 | Retrospective |
| Vrandecic ^8^ | 2002 | 247 | 47.3 | 5.9 | Retrospective |
| **Total** |  | **1677** |  |  |  |
| **Mechanical** | | | | | |
| Weber ^1^ | 2012 | 103 | 50 | 2.7 | Retrospective |
| Bouhout ^9^ | 2013 | 450 | 53 | 9.1 | Retrospective |
| Jamieson ^10^ | 1999 | 384 | 52.3 | 2.5 | Retrospective |
| Nazarov ^11^ | 2014 | 211 | 52.2 | 5.1 | Retrospective |
| Nishida ^2^ | 2014 | 217 | 46.2 | 13.3 | RCT |
| Sakamoto ^12^ | 2005 | 46 | 54.0 | 6.2 | Retrospective |
| Mokhles ^13^ | 2010 | 253 | 48.0 | 6.3 | Retrospective |
| McClure ^3^ | 2014 | 361 | 53.2 | 6.5 | Retrospective |
| Kandemir ^14^ | 2006 | 80 | 46.4 | 6.6 | Retrospective |
| Kandemir ^14^ | 2006 | 94 | 48.8 | 5.8 | Retrospective |
| Andreas ^15^ | 2014 | 173 | 41 | 7.9 | Retrospective |
| Niclauss ^7^ | 2013 | 140 | 53 | 4.7 | Retrospective |
| Calkavur ^16^ | 2002 | 59 | 35.9 | 2.1 | Retrospective |
| Aagaard ^17^ | 2003 | 55 | 33 | 7.6 | Retrospective |
| Ho ^18^ | 2005 | 217 | 31.6 | 5.0 | Retrospective |
| Kang ^19^ | 2005 | 179 | 44.4 | 7.9 | Prospective |
| Emery ^20^ | 2003 | 271 | 40 | 7.2 | Retrospective |
| Waszyrowski ^21^ | 1997 | 81 | 48.7 | 3.7 | Prospective |
| **Total** |  | **3374** |  |  |  |
| **Ross** | | | | | |
| Miskovic ^22^ | 2015 | 209 | 43.1 | 7.9 | Prospective |
| Andreas ^15^ | 2014 | 246 | 29 | 9.0 | Retrospective |
| da Costa ^23^ | 2014 | 414 | 30.8 | 8.2 | Prospective |
| David ^24^ | 2013 | 212 | 34 | 13.8 | Prospective |
| Mastrobuoni ^25^ | 2016 | 306 | 41.7 | 10.0 | Prospective |
| Weimar ^26^ | 2013 | 645 | 42.3 | 8.4 | Prospective |
| Xu ^27^ | 2012 | 58 | 28.3 | 8.2 | Retrospective |
| Escarain ^28^ | 2012 | 253 | 42 | 5.8 | Retrospective |
| Bohm ^29^ | 2009 | 467 | 41 | 5.5 | Retrospective |
| Frigiola ^30^ | 2008 | 110 | 30.2 | 6.7 | Retrospective |
| Klieverik ^31^ | 2007 | 146 | 22.4 | 8.6 | Retrospective |
| Sievers ^32^ | 2006 | 347 | 44.0 | 3.8 | Retrospective |
| El-Hamamsy ^33^ | 2010 | 108 | 38 | 10.2 | RCT |
| Dagenais ^34^ | 2005 | 76 | 51.1 | 5.6 | Prospective |
| Skillington ^35^ | 2013 | 333 | 39.3 | 9.4 | Retrospective |
| Hanke ^36^ | 2010 | 1277 | 41.6 | 5.7 | Retrospective |
| Sievers ^37^ | 2010 | 1620 | 39.2 | 6.6 | Retrospective |
| Elkins ^38^ | 2008 | 487 | 24 | 6.7 | Prospective |
| Chiappini ^39^ | 2007 | 219 | 35.5 | 4.9 | Retrospective |
| Settepani ^40^ | 2005 | 103 | 35.2 | 6.0 | Retrospective |
| Kouchoukos ^41^ | 2004 | 119 | 31 |  | Retrospective |
| Concha ^42^ | 2003 | 169 | 29.9 | 3.0 | Retrospective |
| Total |  | 7924 |  |  |  |

Age expressed as mean age (years); Follow-up expressed as mean follow-up (years);

*RCT*: Randomised control trial;

***SUPPLEMENTAL MATERIAL C REFERENCES***

1. Weber A, Noureddine H, Englberger L, Dick F, Gahl B, Aymard T, Czerny M, Tevaearai H, Stalder M, Carrel TP. Ten-year comparison of pericardial tissue valves versus mechanical prostheses for aortic valve replacement in patients younger than 60 years of age. J Thorac Cardiovasc Surg 2012;**144**(5):1075-83.

2. Nishida T, Sonoda H, Oishi Y, Tatewaki H, Tanoue Y, Shiokawa Y, Tominaga R. Long-term results of aortic valve replacement with mechanical prosthesis or carpentier-edwards perimount bioprosthesis in Japanese patients according to age. Circ J 2014;**78**(11):2688-95.

3. McClure RS, McGurk S, Cevasco M, Maloney A, Gosev I, Wiegerinck EM, Salvio G, Tokmaji G, Borstlap W, Nauta F, Cohn LH. Late outcomes comparison of nonelderly patients with stented bioprosthetic and mechanical valves in the aortic position: a propensity-matched analysis. J Thorac Cardiovasc Surg 2014;**148**(5):1931-9.

4. Bourguignon T, Bouquiaux-Stablo AL, Candolfi P, Mirza A, Loardi C, May MA, El-Khoury R, Marchand M, Aupart M. Very long-term outcomes of the Carpentier-Edwards Perimount valve in aortic position. Ann Thorac Surg 2015;**99**(3):831-7.

5. Forcillo J, El Hamamsy I, Stevens LM, Badrudin D, Pellerin M, Perrault LP, Cartier R, Bouchard D, Carrier M, Demers P. The perimount valve in the aortic position: twenty-year experience with patients under 60 years old. Ann Thorac Surg 2014;**97**(5):1526-32.

6. Une D, Ruel M, David TE. Twenty-year durability of the aortic Hancock II bioprosthesis in young patients: is it durable enough? Eur J Cardiothorac Surg 2014;**46**(5):825-30.

7. Niclauss L, von Segesser LK, Ferrari E. Aortic biological valve prosthesis in patients younger than 65 years of age: transition to a flexible age limit? Interact Cardiovasc Thorac Surg 2013;**16**(4):501-7.

8. Vrandecic M, Fantini FA, Filho BG, de O, da C, Vrandecic E. Long-term results with the Biocor-SJM stentless porcine aortic bioprosthesis. J Heart Valve Dis 2002;**11**(1):47-53.

9. Bouhout I, Stevens LM, Mazine A, Poirier N, Cartier R, Demers P, El-Hamamsy I. Long-term outcomes after elective isolated mechanical aortic valve replacement in young adults. J Thorac Cardiovasc Surg 2013;**148**(4):1341-1346.e1.

10. Jamieson WR, Miyagishima RT, Grunkemeier GL, Germann E, Henderson C, Lichtenstein SV, Ling H, Munro AI. Bileaflet mechanical prostheses for aortic valve replacement in patients younger than 65 years and 65 years of age or older: major thromboembolic and hemorrhagic complications. Can J Surg 1999;**42**(1):27-36.

11. Nazarov VM, Zheleznev SI, Bogachev-Prokophiev AV, Afanasyev AV, Nemchenko EV, Jeltovskiy YV, Lavinyukov SO. CardiaMed mechanical valve: mid-term results of a multicenter clinical trial. Asian Cardiovasc Thorac Ann 2014;**22**(1):9-17.

12. Sakamoto Y, Hashimoto K, Okuyama H, Ishii S, Inoue T, Kinouchi K, Abe T. Carpentier-Edwards pericardial aortic valve in middle-aged patients: comparison with the St. Jude Medical valve. Jpn J Thorac Cardiovasc Surg 2005;**53**(9):465-9.

13. Mokhles MM, Kortke H, Stierle U, Wagner O, Charitos EI, Bogers AJ, Gummert J, Sievers HH, Takkenberg JJ. Survival comparison of the Ross procedure and mechanical valve replacement with optimal self-management anticoagulation therapy: propensity-matched cohort study. Circulation 2010;**123**(1):31-8.

14. Kandemir O, Tokmakoglu H, Yildiz U, Tezcaner T, Cem A, Gunay I, Suzer K, Zorlutuna Y. St. Jude Medical and CarboMedics mechanical heart valves in the aortic position: Comparison of long-term results. Texas Heart Institute Journal 2006;**33**(2):154-159.

15. Andreas M, Wiedemann D, Seebacher G, Rath C, Aref T, Rosenhek R, Heinze G, Eigenbauer E, Simon P, Ruetzler K, Hiesmayr JM, Moritz A, Laufer G, Kocher A. The Ross procedure offers excellent survival compared with mechanical aortic valve replacement in a real-world setting. Eur J Cardiothorac Surg 2014;**46**(3):409-13; discussion 413-4.

16. Calkavur T, Yagdi T, Apaydin A, Islamoglu F, Posacioglu H, Durmaz I, Ozbaran M. Four years' experience with the Edwards-Tekna bileaflet valve prosthesis. J Heart Valve Dis 2002;**11**(2):263-9.

17. Aagaard J, Tingleff J, Andersen PV, Hansen CN. Fourteen years' experience with the CarboMedics valve in young adults with aortic valve disease. J Heart Valve Dis 2003;**12**(1):81-6.

18. Ho THQ, Van P, Phan PK, Pham VN. Up to nine-years' experience with the allcarbon prosthetic heart valve. Journal of Heart Valve Disease 2005;**14**(4):512-517.

19. Kang CH, Ahn H, Kim KH, Kim KB. Long-term result of 1144 CarboMedics mechanical valve implantations. Ann Thorac Surg 2005;**79**(6):1939-44.

20. Emery RW, Erickson CA, Arom KV, Northrup WF, 3rd, Kersten TE, Von Rueden TJ, Lillehei TJ, Nicoloff DM. Replacement of the aortic valve in patients under 50 years of age: long-term follow-up of the St. Jude Medical prosthesis. Ann Thorac Surg 2003;**75**(6):1815-9.

21. Waszyrowski T, Kasprzak JD, Krzeminska-Pakula M, Dziatkowiak A, Zaslonka J. Early and long-term outcome of aortic valve replacement with homograft versus mechanical prosthesis - 8-Year follow-up study. Clinical Cardiology 1997;**20**(10):843-848.

22. Miskovic A, Monsefi N, Karimian-Tabrizi A, Zierer A, Moritz A. A 17-year, single-centre experience with the Ross procedure: fulfilling the promise of a durable option without anticoagulation?dagger. Eur J Cardiothorac Surg 2015;**49**(2):514-9.

23. da Costa FD, Takkenberg JJ, Fornazari D, Balbi Filho EM, Colatusso C, Mokhles MM, da Costa AB, Sagrado AG, Ferreira AD, Fernandes T, Lopes SV. Long-term results of the Ross operation: an 18-year single institutional experience. Eur J Cardiothorac Surg 2014;**46**(3):415-22; discussion 422.

24. David TE, Armstrong S, Maganti M. Hancock II bioprosthesis for aortic valve replacement: the gold standard of bioprosthetic valves durability? Ann Thorac Surg 2010;**90**(3):775-81.

25. Mastrobuoni S, de Kerchove L, Solari S, Astarci P, Poncelet A, Noirhomme P, Rubay J, El Khoury G. The Ross procedure in young adults: over 20 years of experience in our Institution. Eur J Cardiothorac Surg 2016;**49**(2):507-12; discussion 512-3.

26. Weimar T, Charitos EI, Liebrich M, Roser D, Tzanavaros I, Doll N, Hemmer WB. Quo vadis pulmonary autograft--the ross procedure in its second decade: a single-center experience in 645 patients. Ann Thorac Surg 2013;**97**(1):167-74.

27. Xu Z, Li W, Xu X, Zhou Z, Song S, Ma J, Zhang J. Long-term follow-up with Ross procedure at a single institution in China. Thorac Cardiovasc Surg 2012;**62**(3):216-21.

28. Escarain MC, Bozovich GE, Salvatori C, Favaloro RR. The ross procedure: A fifteen-year experience [English;Spanish] Cirugia de ross: 15 anos de experiencia. Revista Argentina de Cardiologia 2012;**80**(5):347-353.

29. Bohm JO, Hemmer W, Rein JG, Horke A, Roser D, Blumenstock G, Botha CA. A single-institution experience with the Ross operation over 11 years. Ann Thorac Surg 2009;**87**(2):514-20.

30. Frigiola A, Ranucci M, Carlucci C, Giamberti A, Abella R, Di M. The Ross Procedure in Adults: Long-Term Follow-Up and Echocardiographic Changes Leading to Pulmonary Autograft Reoperation. Annals of Thoracic Surgery 2008;**86**(2):482-489.

31. Klieverik LM, Takkenberg JJ, Bekkers JA, Roos-Hesselink JW, Witsenburg M, Bogers AJ. The Ross operation: a Trojan horse? Eur Heart J 2007;**28**(16):1993-2000.

32. Sievers HH, Hanke T, Stierle U, Bechtel MF, Graf B, Robinson DR, Ross DN. A critical reappraisal of the Ross operation: renaissance of the subcoronary implantation technique? Circulation 2006;**114**(1 Suppl):I504-11.

33. El-Hamamsy I, Eryigit Z, Stevens LM, Sarang Z, George R, Clark L, Melina G, Takkenberg JJ, Yacoub MH. Long-term outcomes after autograft versus homograft aortic root replacement in adults with aortic valve disease: a randomised controlled trial. Lancet 2010;**376**(9740):524-31.

34. Dagenais F, Cartier P, Voisine P, Desaulniers D, Perron J, Baillot R, Raymond G, Metras J, Doyle D, Mathieu P. Which biologic valve should we select for the 45- to 65-year-old age group requiring aortic valve replacement? J Thorac Cardiovasc Surg 2005;**129**(5):1041-9.

35. Skillington PD, Mokhles MM, Takkenberg JJ, O'Keefe M, Grigg L, Wilson W, Larobina M, Tatoulis J. Twenty-year analysis of autologous support of the pulmonary autograft in the Ross procedure. Ann Thorac Surg 2013;**96**(3):823-9.

36. Hanke T, Charitos EI, Stierle U, Robinson DR, Hemmer W, Moritz A, Lange R, Sievers HH. The Ross operation - a feasible and safe option in the setting of a bicuspid aortic valve? Eur J Cardiothorac Surg 2010;**38**(3):333-9.

37. Sievers HH, Stierle U, Charitos EI, Hanke T, Misfeld M, Matthias Bechtel JF, Gorski A, Franke UF, Graf B, Robinson DR, Bogers AJ, Dodge-Khatami A, Boehm JO, Rein JG, Botha CA, Lange R, Hoerer J, Moritz A, Wahlers T, Breuer M, Ferrari-Kuehne K, Hetzer R, Huebler M, Ziemer G, Takkenberg JJ, Hemmer W, German-Dutch Ross R. Major adverse cardiac and cerebrovascular events after the Ross procedure: a report from the German-Dutch Ross Registry. Circulation 2010;**122**(11 Suppl):S216-23.

38. Elkins RC, Thompson DM, Lane MM, Elkins CC, Peyton MD. Ross operation: 16-year experience. J Thorac Cardiovasc Surg 2008;**136**(3):623-30, 630.e1-5.

39. Chiappini B, Absil B, Rubay J, Noirhomme P, Funken JC, Verhelst R, Poncelet A, El Khoury G. The Ross procedure: clinical and echocardiographic follow-up in 219 consecutive patients. Ann Thorac Surg 2007;**83**(4):1285-9.

40. Settepani F, Kaya A, Morshuis WJ, Schepens MA, Heijmen RH, Dossche KM. The Ross operation: an evaluation of a single institution's experience. Ann Thorac Surg 2005;**79**(2):499-504.

41. Kouchoukos NT, Masetti P, Nickerson NJ, Castner CF, Shannon WD, Davila-Roman VG. The Ross procedure: long-term clinical and echocardiographic follow-up. Ann Thorac Surg 2004;**78**(3):773-81; discussion 773-81.

42. Concha M, Pradas G, Juffe A, Caffarena JM, Montero A, Aranda PJ. Comprehensive experience with the Ross operation in Spain. Eur J Cardiothorac Surg 2003;**24**(4):521-6.
